# Molecular phylogeny of selected dorid nudibranchs based on complete mitochondrial genome

**DOI:** 10.1038/s41598-022-23400-9

**Published:** 2022-11-05

**Authors:** Thinh Dinh Do, Dae-Wui Jung, Chang-Bae Kim

**Affiliations:** 1 Department of Biotechnology, Sangmyung University, Seoul, 03016 South Korea; 2 Institute of Marine Environment and Resources, Vietnam Academy of Science and Technology, Haiphong, 04000 Vietnam

**Keywords:** Evolution, Genetics, Systems biology

## Abstract

Dorid nudibranchs are a large group of mollusks with approximately 2,000 recorded species. Although agreement exists on the monophyletic nature of the dorid nudibranch group, the interfamily relationships of the suborder are subject to debate. Despite efforts to elucidate this issue using short molecular markers, the conclusiveness of the findings has been hindered by branching polytomy. Mitogenomes are known to be effective markers for use in phylogenetic investigations. In this study, eight mitogenomes of dorid nudibranchs were decoded and analyzed. Gene content and structure showed little change among species, reflecting the conserved mitogenomes of dorid nudibranchs. For most genes, the direction was typical for nudibranchs; nevertheless, tRNA^Cys^ had an inverse direction in Cadlinidae species. Phylogenetic trees based on nucleotide and amino acid datasets revealed a relatively consistent pattern of interfamily relationships with little difference for positions of Phyllidiidae and Cadlinidae. Species of Cadlinidae were clustered together and did not form a clade with Chromododidae. Additionally, Goniodorididae was sister to Aegiridae, whereas Discodoridae was sister to Dorididae. This finding was supported by tree topology test based on mitogenome data. The results of the present study indicate that complete mitogenomes are promising markers for investigating interfamily relationships among dorid nudibranchs.

## Introduction

Doridina (~ 2,000 species) and its sister group Cladobranchia (~ 1,000 species) are two suborders of mollusk nudibranchs^1,2^. The dorid nudibranchs are a diverse group of marine mollusks found worldwide that play an important role in the marine ecosystem. Dorid species are carnivorous; they feed mainly on sedentary invertebrates such as sponges, cnidarians, tunicates, and bryozoans^3^. To deter their own predators, many dorid species synthesize unpleasant or toxic compounds from their foods^3^, this ability makes dorid nudibranchs potentially interesting subjects in the search for chemical compounds with pharmaceutical relevance^4^. Thus, several species have been used in pharmaceutical science and developmental studies. *Cadlina luteomarginata* has been of particular interest in biochemical investigations, whereas other nudibranch species, such as *Aldisa andersoni*, *Aldisa cooperi*, *Cadlina pellucida*, *Cadlina laevis*, *Doriprismatica atromarginata*, and *Jorunna funebris*, have become important subjects in bioactive substance studies^3–7^. However, the first step in the practical application of dorid nudibranch compounds is the elucidation of the group’s taxonomy and phylogenetic relationships.

Despite their importance in marine ecology and pharmaceutical studies, the interfamily relationships of dorid nudibranchs have long been disputed^8^. Previously, only the morphological characteristics of dorid families, such as the rhinophores, mantle, gill, gill cavity, and radula, were used for classification; however, molecular markers are now used to study dorid nudibranch phylogeny^9,10^. In such studies, a single marker or combination of several short markers is usually used. Although some studies have been conducted to determine relationships within a genus or family, only a few studies have dealt with the higher-level groups in Doridina and Cladobranchia^2,11^. Moreover, the application of short markers has been difficult to elucidate the phylogenies containing these higher-level groups. To date, few attempts have been made to study the interfamily relationships of dorid nudibranchs using cladistic methods. Notable research has been published by Hallas et al.^11^ and Korshunova et al.^8^ related to the families within the suborder Doridina. Nevertheless, interfamily relationships remain poorly understood because of conflicting phylogenies, tree polytomy, and inadequate sampling^11^. For example, controversy surrounds the relationships between Discodoridae + Dorididae and Goniodorididae + Aegiridae. Conventionally, Discodoridae was considered to have a close relationship with Dorididae, whereas Goniodorididae was believed to have a close relationship with Aegiridae^12^. Nevertheless, recent molecular analyses have shown that Discodoridae is a sister group to Goniodorididae, whereas Aegiridae is a sister group to Dorididae^8^ or has an unstable position (depending on the analysis method used)^11^. To improve the systematics of dorid nudibranchs, phylogenetic relationships must be explicitly determined, and the effective use of DNA sequences to elucidate phylogenies is one potential strategy. Another issue is the phylogenetic classification of Cadlinidae, which has long been controversial. Traditionally, Cadlinidae has been considered a member of Chromodorididae; however, recent taxonomic evaluation indicated that Cadlinidae is an independent family that is separate from Chromodorididae^8,13^.

The mitochondrial genome is a powerful molecular marker used to explore phylogenetic relationships, and it has been applied to reveal the molecular evolution of mollusks^14–16^. The typical mitogenome of mollusks contains 13 protein-coding genes, 22 transfer RNA (tRNA) genes, and two ribosomal RNA (rRNA) genes^14^. Given their importance in systematics and phylogenetic reconstruction, the mitogenomes of nudibranchs are now being characterized; however, too few of the mitogenomes from more than 2,000 dorid nudibranchs have been sequenced and analyzed. Molecular phylogenetic analyses and taxon-sampling schemes are known as effective tools in phylogenetic research^13^. Previously, the phylogenetic position of nudibranchs has been studied on the basis of partial *cox1*, 16S rRNA, 18S rRNA, and 28S rRNA sequences. Nevertheless, the complete mitogenome provides better phylogenetic resolution and accuracy than single genetic markers^17^.

This study aimed to analyze the mitogenome structure of dorid nudibranchs and use mitogenomes as molecular markers to investigate the interfamily relationships of this group. To achieve this aim, the mitogenomes of different dorid nudibranchs were decoded and analyzed. The structure of dorid nudibranch mitogenomes was examined and compared with the mitogenome sequences already available in public databases. Additionally, phylogenetic trees showing interfamily relationships were determined on the basis of the examined dorid nudibranch mitogenomes.

## Results

### General mitgenome features

Eight complete mitogenomes of dorid nudibranchs were sequenced in the present study, including those of *Aldisa cooperi*, *Cadlina japonica*, *Cadlina koreana*, *Cadlina umiushi*, *Carminodoris armata*, *Doris odhneri*, *Triopha modesta*, and *Verconia nivalis*. Mitogenome lengths ranged from 14,397 bp (*T. modesta*) to 14,982 bp (*C. japonica*) (Tables S1–8; Figs. S1–8). All eight mitogenomes had negative AT skew values (from − 0.167 in *V. nivalis* to − 0.089 in *D. odhneri*) and positive GC skew values (from 0.008 in *D. odhneri* to 0.152 in *Cadlina umiushi*), suggesting a bias for T and G nucleotides (Table S9).Generally, mitogenomes contained 13 protein-coding genes, two rRNA genes, and 22 tRNA genes. The mitogenomes of most species comprised 37 genes; however, that of *C. japonica* contained 38 genes due to a duplication of tRNA^Ile^. The mitogenome of each species contained 13 protein-coding genes (PCGs), including nine genes (*cox1*, *cox2*, *cytb*, *nd1*, *nd2*, *nd4*, *nd4l*, *nd5*, and *nd6*) encoded by the H-strand and four genes (*atp6*, *atp8*, *cox3*, and *nd3*) encoded by the L-strand (Tables S1–8). In terms of start and stop codons, ATN was the most frequent initiation codon, ATG was most commonly used, and ATA, GTG, and TTG were also used for initiation; TAA was the most common termination codon, with TAG and incomplete T– were also used for the termination of several genes. Codon usage and relative synonymous codon usage (RSCU) are indicated in Table S10. For all mitogenomes, the amino acids most frequently found in PCGs were leucine followed by serine; by contrast, glutamine, arginine and cysteine were the least common amino acids. The RSCU values of 13 PCGs in the eight examined mitogenomes showed a bias toward amino acids encoded by codons rich in A and T, such as UUA-Leu, AUU-Ile, UUU-Phe, and AUA-Met (Table S10).

There were 22 tRNA genes in most dorid species, except the *C*. *japonica* mitogenome carried 23 genes due to a tRNA^Ile^ duplication. Similar to other nudibranchs, different anticodons were observed for tRNA^Leu^ and tRNA^Ser^. Two rRNA genes were detected in the mitogenomes of dorid nudibranchs. The large (16S rRNA) and small (12S rRNA) rRNAs were encoded by the H-strand and L-strand, respectively. Overall, the intergenic regions in the eight dorid nudibranch mitogenomes were short in length. In the present study, the sizes of the intergenic regions varied according to species. The longest noncoding region was located between tRNA^His^ and tRNA^Cys^ in Cadlinidae species (324 bp in *C. japonica*). The overlapping regions were also short and variable among species; nevertheless, the longest overlapping region was always located between *nd5* and *nd1* genes.

**Figure 1:**
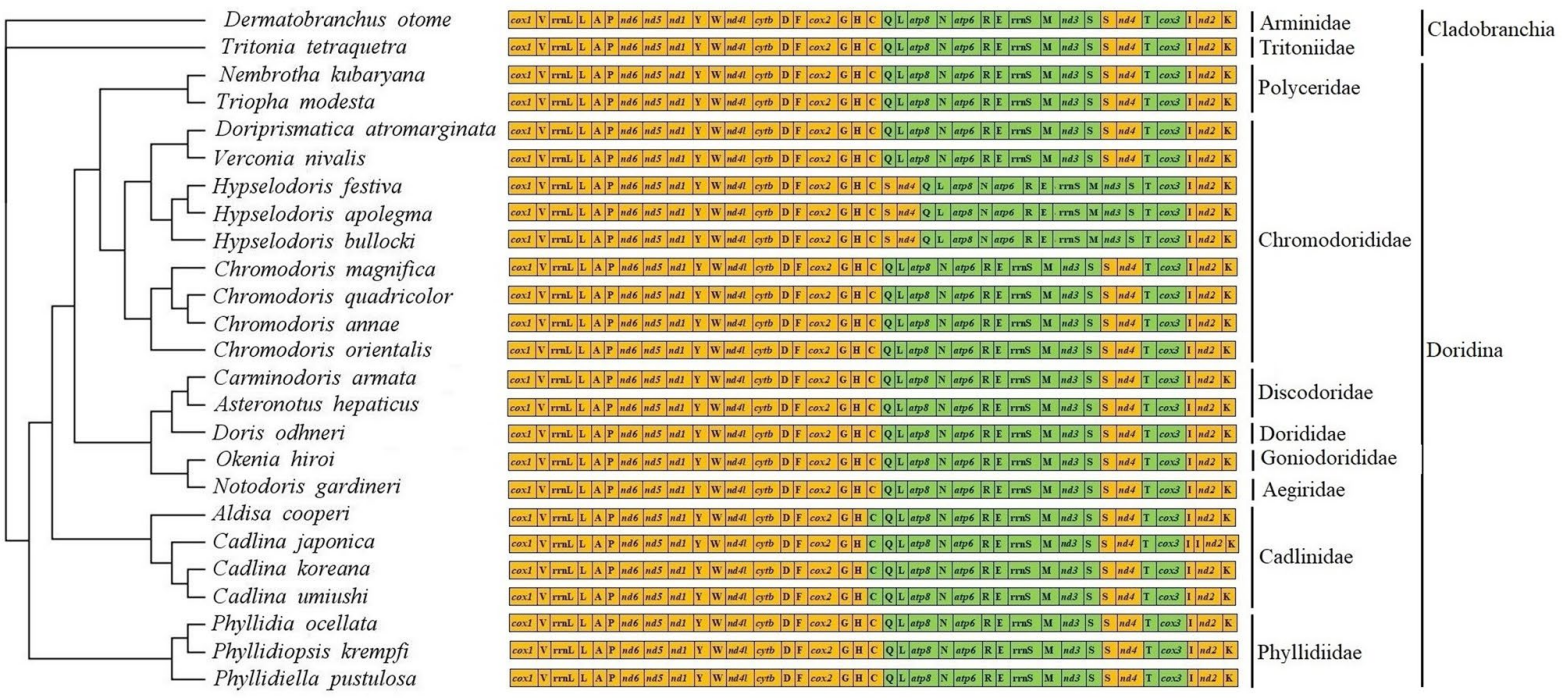
Linearized mitochondrial gene arrangement patterns of the suborders Doridina and Cladobranchia. Species relationships were based on phylogenetic analyses presented in Fig. 2. Yellow and green represent genes encoded on the H-strand and L-strand, respectively. Single-letter abbreviation of the amino acid code represents tRNAs.

The gene contents and order were similar to those in typically structured Nudibranchia mitogenomes. Gene direction was similar across the examined mitogenomes, although the direction of tRNA^Cys^ was inverse in species from Cadlinidae, i.e., *A. cooperi*, *C. japonica*, *C. koreana*, and *C. umiushi*, compared with its direction in other nudibranchs (Fig. 1). In the four Cadlinidae species, tRNA^Cys^ was encoded by the L-strand; in the other nudibranchs, it was encoded by the H-strand. Overall, gene order within Doridina mitogenomes is pretty conservative and identical to that of arrangement pattern in Nudibranchia. An exception was observed in the mitogenomes of *Hypselodoris* which present translocation of the second tRNA^Ser^ (GCU) and *nd4*^18,19^. In most nudibranch, the second tRNA^Ser^ and *nd4* are located between the first tRNA^Ser^ and tRNA^Thr^. However, in three recorded *Hypselodoris* species, this block is translocated to the position between tRNA^Cys^ and tRNA^Gln^ (Fig. 1).

### Phylogeny of dorid nudibranchs based on complete mitogenome sequences

**Figure 2:**
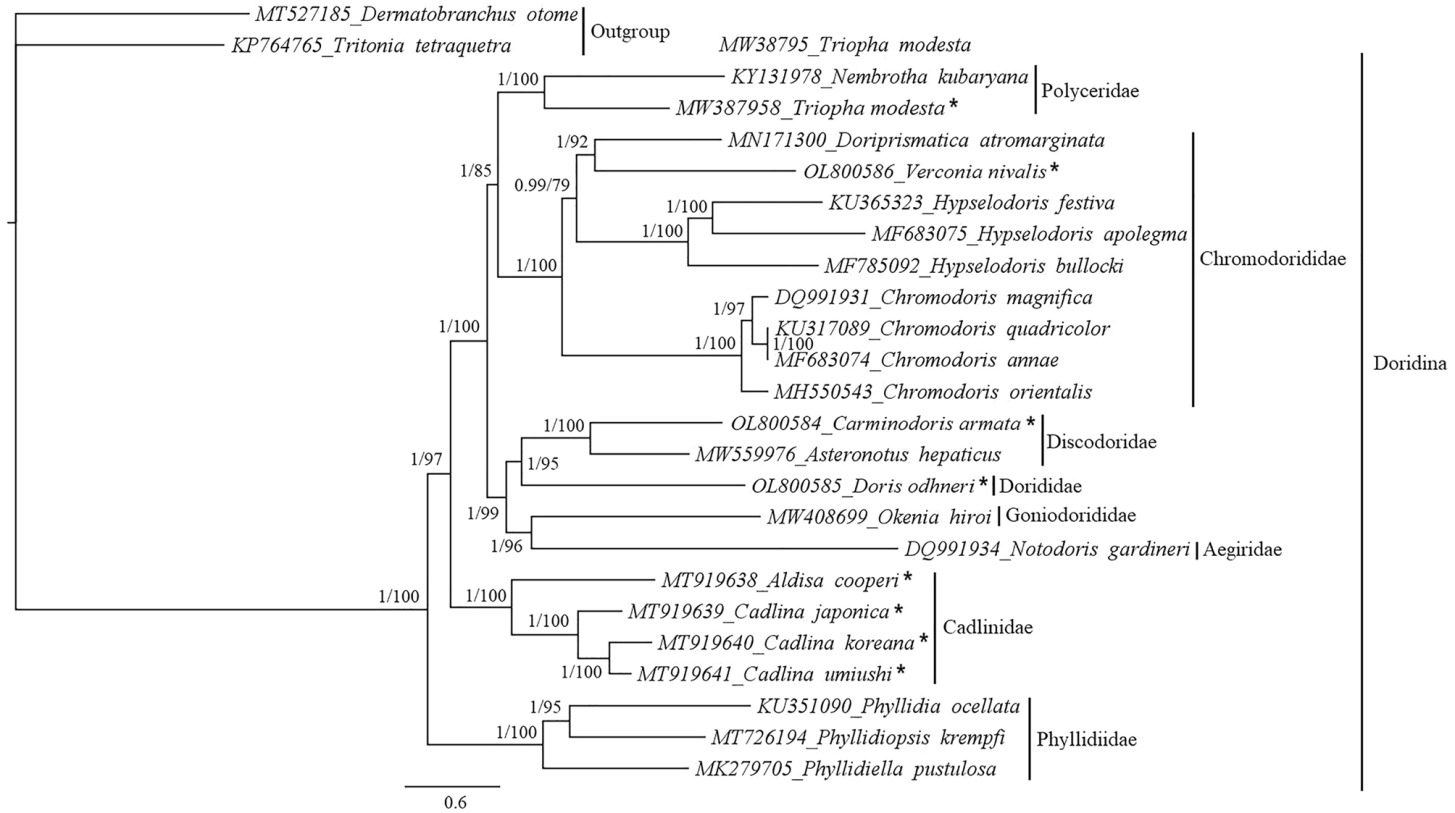
Phylogenetic tree showing the interfamily relationships of dorid nudibranchs based on the nucleotide sequences of 12 PCGs + 2 rRNAs + 22 tRNAs from mitogenomes (*nd4l* excluded). Sequences generated in this study are marked with stars. GenBank accession numbers are indicated next to species names. Gblocks was used after sequence alignment. Posterior possibility values (left) and ultrafast bootstrap values (right) are shown at the nodes. Species of the suborder Cladobranchia were used as outgroup.

Phylogenetic trees of dorid nudibranchs were constructed with and without Gblocks based on nucleotide and amino acid datasets of mitogenome sequences. For each dataset, two tree construction methods, Bayesian inference (BI) and Maximum likelihood (ML), were used. Because the BI and ML analyses showed similar phylogenetic topologies for all datasets, the trees were combined to show the interfamily relationships of the dorid nudibranchs (Fig. 2; Figs. S9–15). The use of three outgroup species revealed little change in tree reliability compared to two outgroup species (Fig. S16-S19). As shown in phylogenetic trees, the interfamily relationships were well resolved. In general, phylogenetic trees indicated high posterior priority (PP) values, while ultrafast bootstrap (UFBoot) values were variable among datasets. High credibility was observed in tree generated based on the nucleotide sequences of 12 PCGs + 2 rRNAs + 22 tRNAs with Gblocks. BI tree of this dataset showed PP ≥ 0.99, meaning that polytomy is not observed if threshold < 0.99. For interfamily relationship in ML tree, the lowest support value was found between Polyceridae and Chromodorididae (UFBoot = 85). Polytomy of this node occurs if threshold is set as 85, while UFboot values for other family relationships show strong supports (Fig. 2).

All examined families were recovered as monophyly in the phylogenetic trees generated in this study. Moreover, the branching patterns for interfamily relationships in the trees were similar except trees generated from 1st and 2nd codon dataset. In most trees, three species of Phyllidiidae were branched early, while in 1st and 2nd codon tree, the members of Cadlinidae were branched early. Expectedly, three *Cadlina* species (*C*. *japonica*, *C*. *koreana*, and *C*. *umiushi*) were clustered together with absolute support values for both PP and UFBoot in all analyzed trees (PP = 1; UFBoot = 100). The clade containing these three *Cadlina* species was a sister to *A. cooperi* (PP = 1; UFBoot = 100). The phylogenetic trees also showed that Goniodorididae was a sister group to Aegiridae, whereas Discodoridae was a sister group to Dorididae. Support values for these relationships were variable among analyses. In a tree based on the nucleotide sequences of 12 PCGs + 2 rRNAs + 22 tRNAs with Gblocks, support values were as follows: Discodoridae + Dorididae: PP = 1 and UFBoot = 95; Aegiridae + Goniodorididae: PP = 1 and UFBoot = 96 (Fig. 2). Additionally, these two clades were clustered together with high support values (PP = 1; UFBoot = 99). The amino acid tree showed a similar pattern of interfamily relationships but lower support values relative to those of the nucleotide tree. In a tree based on 12 amino acid sequences with Gblocks, support values were as follows: Discodoridae + Dorididae: PP = 0.99 and UFBoot = 73; Aegiridae + Goniodorididae: PP = 0.98 and UFBoot = 65 (Fig. S14). The clade containing these four families was a sister to a clade that included Chromodorididae and Polyceridae. Although the relationships among the four Chromodorididae genera were slightly variable among datasets, this family was always a sister to Polyceridae in all generated trees.

**Figure 3:**
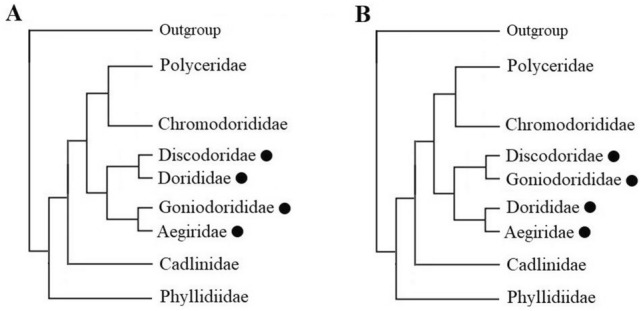
Interfamily relationship from this study (**A**) and constrained branching pattern (**B**) used for topology test. In the constrained tree, Discodoridae is sister to Goniodoridae and Aegiridae is sister to Dorididae. The families targeted for topology test were marked with black circles.

A tree topology test was performed to investigate the relationships among Discodoridae, Dorididae, Goniodorididae, and Aegiridae. The tree topology and constrained tree pattern are shown in Fig. 3. The topology of the tree from the present study was confirmed as the most likely dorid nudibranch phylogeny (*p* = 0.974, i.e., *p* > 0.05), whereas the topology of the constrained tree was rejected (*p* = 0.026, i.e., *p* < 0.05). Therefore, the relationships of Discodoridae + Dorididae and Aegiridae + Goniodorididae were statiscally supported, whereas those of Aegiridae + Dorididae and Goniodorididae + Discodoridae were rejected.

## Discussion

Despite the rich diversity of dorid nudibranchs, a limited number of their mitogenomes are recorded in public databases; this hinders the use of mitogenomes in investigations of dorid nudibranch evolution and phylogeny. In the present study, mitogenomes from different families of dorid nudibranchs were sequenced and characterized. Generally, these mitogenomes were small (14,397–14,982 bp); thus, they were similar in size to those of other gastropods. The small mitogenome size is attributable to the low number of noncoding regions, the overlap of genes, and the reduced size of genes in all sequences^14^. Base skewness was also consistent among the studied species (negative A-T skew and positive G-C skew), and gene arrangement was similar to that recorded in other nudibranch mitogenomes. Among the PCGs in the mitogenomes, codons were typically rich in A and T. Generally, the gene arrangement of nudibranchs is conserved with little variation. Indeed, a change in gene arrangement has only been observed in *Hyselodoris*^18,19^. Accordingly, tRNA^Ser^ and *nd4* were located between tRNA^Cys^ and tRNA^Gln^. The prominent difference observed was the inverse direction of tRNA^Cys^ in all four Cadlinidae species. This characteristic was not found in the other nudibranchs studied here, but it has been observed in *Berthella* sp. from Pleurobranchida, which is the sister order of Nudibranchia^20^. This change might have specifically occurred during evolution. Except for the inverse direction of tRNA^Cys^ in Cadlinidae, the gene direction in the studied dorid species was typical of other nudibranchs. We also identified the duplication of tRNA^Leu^ in *C. japonica*, which is the first time a gene duplication has been observed in a dorid nudibranch.

Huge efforts have been made to investigate interfamily relationships of dorid nudibranchs^8–11^. Recently, molecular markers^11^ or a combination of molecular markers and morphological characteristics^8^ were applied for this purpose. Although short markers, such as partial *cox1* and 16S rRNA genes, have previously been used to build phylogenetic trees for dorid nudibranchs, satisfactory conclusions could not be drawn regarding interfamily relationships due to polytomy and instability of family branching^8,11^. In the present study, we attempted to address this issue using mitogenomes as markers. Despite the small number of mitogenome sequences used in this study relative to the total number of dorid nudibranch families, our findings are valuable, and promising. First, the branching of trees based on different datasets was pretty clear and consistent. Comparing tree topology among datasets, little difference in branching pattern was observed when first and second codons of PCGs were used. The trees based on this dataset showed Cadlinidae early branched instead of Phyllidiidae (Fig. S12-13). However, PP and UFBoot values between Cadlinidae and Phyllidiidae with inside branch were not high. This may be caused by the lack of samples for the whole suborder Doridina. Therefore, increase in sample coverage for different families is necessary to gain an insight into phylogeny of dorid nudibranch.

Through phylogenetic analyses, significant interfamily relationships were detected for the suborder Doridina. Congruent with previous studies, our results revealed the existence of a cluster containing Polyceridae and Chromodorididae^8,11^. Importantly, our phylogenetic trees showed that Discodoridae was a sister group to Dorididae and that these two families were a sister group to a clade containing Goniodorididae and Aegiridae. The identified relationships among these families are not consistent with those reported in previous studies^8,11^. However, our findings are congruent with morphological evidence because Discodoridae and Dorididae possess characteristics of cryptobranchs, whereas Goniodorididae and Aegiridae possess characteristics of phanerobranchs^8,10^. Additionally, the (Aegiridae + Goniodorididae) + (Discodoridae + Dorididae) was in agreement with conventional classification. It is widely accepted that dorid nudibranchs can be divided into two groups: cryptobranchia (with a gill cavity, e.g., Dorididae and Discodoridae) and phanerobranchia (without a gill cavity, e.g., Aegiridae and Goniodorididae)^8^. Moreover, our topology test based on mitogenome sequences significantly supported this pattern and rejected the sister relationship between Discodoridae and Goniodorididae as well as that between Aegiridae and Dorididae.

Additionally, our study supported the separation of Cadlinidae from Chromodorididae. Historically, the classification of Chromodorididae was based on morphological similarities, primarily radular and reproductive morphology, and *Cadlina* was thought to be closely related to members of Chromodorididae such as *Chromodoris* and *Hypselodoris*^13^. However, besides shared characteristics, some of the characteristics possessed by *Cadlina* are not present in Chromodorididae. For example, contrary to chromodorids, *Cadlina* possesses penial spines, spicules in the mantle tissues, and tubercles on the mantle surface^8,13^. Similar to *Cadlina*, the taxonomic position of *Aldisa* has been disputed. Nevertheless, the denticulate teeth of the radular suggest that both genera are associated with chromodorids^8,13^. By contrast, some characteristics are shared by *Cadlina* and *Aldisa*, e.g., the tuberculate mantle and differentiated stomach, whereas these characteristics are not found in chromodorids^13^. Besides morphology, the geographical distribution differs between Cadlinidae and Chromodorididae: Chromodorididae is commonly found in tropical and subtropical waters, whereas Cadlinidae is distributed in temperate and cold waters^8^. Unlike the disputed morphological characteristics, molecular evidence suggests that Cadlinidae is distant from Chromodorididae^13,21^. Besides the morphological evidence, a phylogeny based on *cox1* and 16S rRNA markers indicated that Cadlinidae was a distinct family^13^. Following a study by Jonhson^13^, additional molecular evidence has been accumulated and analyzed. Koroshunova et al.^8^ recently investigated the relationships among dorid nudibranchs, including Cadlinidae and Chromodorididae, by concatenating *cox1* + 16S rRNA + 18S rRNA + 28S rRNA sequences; they also showed the distinct separation of Cadlinidae. Hence, consistent with previous reports, our phylogenetic trees based on mitogenome data confirmed that Cadlinidae is a distinct family.

## Conclusion

In this study, eight new mitogenomes of dorid nudibranchs were characterized and compared with previously recorded mitogenomes from this suborder. Little change in gene content and structure revealed the conserved mitogenome of dorid nudibranchs. Variation in gene direction was only observed in Cadlinidae with the inversion of tRNA^Cys^. We have provided the most comprehensive phylogeny of dorid nudibranchs to date based on mitogenomes. From analyses of nucleotide and amino acid datasets, we revealed a pretty consistent pattern of branching among interfamily relationships. Well branched phylogeny revealed that complete mitogenomes are promising markers for investigating the phylogenies of dorid nudibranchs. Despite our promising results, we were unable to cover all families of dorid nudibranchs. To better understand the overall phylogeny of this group, additional mitogenome sequences from different families should be sequenced and analyzed. Moreover, nuclear genes and transcriptomic data should be used to provide better phylogenetic resolution.

## Methods

### Sample collection and mitogenome sequencing

Specimens were collected from different localities in South Korea during scuba diving sessions (Table S11). Upon collection, samples were preserved in 95% ethanol in preparation for DNA extraction. Before starting mitogenome sequencing, all species were identified using DNA barcoding (data not shown). Following species confirmation, total DNA was extracted from the feet of the specimens using E.Z.N.A.® Mollusk DNA Kit (Omega Bio-tek, Norcross, USA). Library preparation was conducted using Illumina TruSeq Nano DNA Sample Prep Kit (Illumina, San Diego, USA) following the manufacturer’s instructions. Paired-end reads of mitogenomes were generated from prepared libraries using an Illumina MiSeq system (Illumina, San Diego, USA). First, data quality was checked, and clean reads were generated by trimming adapters and low-quality bases. Subsequently, MITObim was used to assemble the mitogenome sequences from clean reads^22^. Fragments of *cox1* from the same species were used as bait for assembly. Mitogenome sequences were annotated on the MITOS web server using the invertebrate genetic code^23^.

PCGs and rRNA genes were aligned with homologous genes from other nudibranchs and confirmed using BLAST searches in GenBank. Additionally, tRNA structures were predicted and identified using the MITOS web server^23^ and ARWEN^24^. Circular maps of complete mitogenomes were generated and annotated using Geneious v9.1^25^. Skewness was assessed using the following formulas: AT skew = [A − T] / [A + T]; GC skew = [G − C] / [G + C]^26^. RSCU values were calculated using MEGA X to evaluate the level of nucleotide bias in each codon^27^.

### Phylogenetic analysis

The mitogenomes generated in this study and those obtained from GenBank for other nudibranchs were used for phylogenetic analyses (Table 1). Two species of the suborder Cladobranchia, *Dermatobranchus otome* and *Tritonia tetraqueta* were used as outgroup. Both amino acid and nucleotide sequences were applied to construct phylogenetic trees. Because *nd4l* gene of *Notodoris gardineri* was half as short as that of other species, this gene was excluded from the analyses. For both amino acid and nucleotide sequences, each sequence was extracted and aligned using MAFFT v7^40^ in Geneious v9.1^25^. To check the impact of the variable regions of mitogenomes on phylogenetic trees, two alignment schemes were used^11^. In the first scheme, following alignment, sequences were directly concatenated without the use of Gblocks. In the second scheme, Gblocks v0.91b was used to remove poorly aligned regions^41^. Four datasets were used to build the phylogenetic trees: nucleotide sequences of 12 PCGs + 2 rRNAs + 22 tRNAs, nucleotide sequences of 12 PCGs, 1st and 2nd codons of 12 PCGs and amino acid sequences of 12 PCGs. For the nucleotide and amino acid sequences of the 12 PCGs, each of the 12 sequences was set for a separate partition. The sequences of each mitogenome were concatenated using Geneious v9.1^25^. The best partition scheme and the best fit model were determined using Partition Finder 2^42^. For a dataset with 36 nucleotide sequences, each of the 12 PCGs and two rRNA genes were set for separate partitions and the 22 tRNA genes were set for a partition. Also, for testing the impact of outgroup on phylogenetic reliability, different outgroup including three species of the suborder Cladobranchia, *Melibe leonine*, *Protaeolidiella atra* and *Sakuraeolis japonica* were used to compared BI and UFboot values among trees. Data fore phylogenetic analyses were prepared as description above with the use of Gblocks^41^.

**Table 1:** Complete mitogenomes used in this study. Species names and systematics are used following the World Register of Marine Species at http://www.marinespecies.org.

Family	Species	Length (bp)	Genbank no	References
Cadlinidae	*Aldisa cooperi*	14,517	MT919638	This study
Cadlinidae	*Cadlina japonica*	14,982	MT919639	This study
Cadlinidae	*Cadlina koreana*	14,707	MT919640	This study
Cadlinidae	*Cadlina umiushi*	14,731	MT919641	This study
Dorididae	*Doris odhneri*	14,445	OL800585	This study
Discodorididae	*Carminodoris armata*	14,424	OL800584	This study
Discodorididae	*Asteronotus hepaticus*	14,464	MW559976	Unpublished
Polyceridae	*Triopha modesta*	14,397	MW387958	This study
Polyceridae	*Nembrotha kubaryana*	14,395	KY131978	Xiang et al.^28^
Chromodorididae	*Verconia nivalis*	14,595	OL800584	This study
Chromodorididae	*Chromodoris magnifica*	14,446	DQ991931	Medina et al.^17^
Chromodorididae	*Chromodoris quadricolor*	14,259	KU317089	Xiang et al.^29^
Chromodorididae	*Chromodoris annae*	14,260	MF683074	Lin et al.^30^
Chromodorididae	*Chromodoris orientalis*	14,260	MH550543	Yu et al.^31^
Chromodorididae	*Hypselodoris festiva*	14,880	KU365323	Karagozlu et al.^18^
Chromodorididae	*Hypselodoris apolegma*	14,749	MF683075	Lin et al.^19^
Chromodorididae	*Hypselodoris bullocki*	14,666	MF785092	Lin et al.^19^
Chromodorididae	*Doriprismatica atromarginata*	14,421	MN171300	Do et al. 2019^32^
Phyllidiidae	*Phyllidia ocellata*	14,598	KU351090	Xiang et al.^33^
Phyllidiidae	*Phyllidiella pustulosa*	14,717	MK279705	Do et al.^34^
Phyllidiidae	*Phyllidiopsis krempfi*	14,970	MT726194	Kim et al.^35^
Aegiridae	*Notodoris gardineri*	14,424	DQ991934	Medina et al.^17^
Goniodorididae	*Okenia hiroi*	14,606	MW408699	Do et al.^36^
Arminidae	*Dermatobranchus otome*	14,559	MT527185	Do et al.^37^
Tritoniidae	*Tritonia tetraqueta*	14,540	KP764765	Sevigny et al.^14^
Tethydidae	*Melibe leonina*	14,513	KP764764	Sevigny et al.^14^
Pleurolidiidae	*Protaeolidiella atra*	14,445	MN911169	Do et al.^38^
Facelinidae	*Sakuraeolis japonica*	15,059	KX610997	Karagozlu et al.^39^

The phylogenetic trees were constructed with Maximum Likelihood (ML) and Bayesian Inference (BI) methods. ML trees was searched using IQ-tree v.2.1.2 with 1,000 bootstrap replicates^43^. BI trees were searched using MrBayes v3.2.7 with four chains and 20,000,000 and 3,000,000 generations for the nucleotide dataset and amino acid dataset, respectively^44^. Additionally, sampling was performed every 100 generations and 25% of the first tree was set as burn-in. Each run was checked for proper mixing and convergence on the basis of ESS values of > 200 in Tracer v1.7^45^. The maximum clade credibility tree was visualized using FigTree v1.4.4^46^.

A tree topology test was performed using the mitogenome sequences and IQ-tree v2.1.2, to compare the interfamily relationships of dorid nudibranchs found in the present study with those found in previous reports^43^. The tree topology from the present study was tested against a constrained tree in which Aegiridae was a sister group to Dorididae and Goniodorididae was a sister group to Discodoridae. *P*-values for approximately unbiased tests were obtained from IQ-tree v2.1.2 using 20,000 bootstrap replicates^43^.

## Supplementary Information

Supplementary Information 1.

Supplementary Information 2.

## Data Availability

The mitogenome sequences generated during the current study are available in GenBank under accession numbers: *Aldisa cooperi* (MT919638), *Cadlina japonica* (MT919639), *Cadlina koreana* (MT919640), *Cadlina umiushi* (MT919641), *Carminodoris armata* (OL800584), *Doris odhneri* (OL800585), *Triopha modesta* (MW387958), and *Verconia nivalis* (OL800586).
