# Supplementary material for: Molecular phylogeny of selected dorid nudibranchs based on complete mitochondrial genome
Source: Sci Rep. 2022 Nov 5;12:18797. doi: 10.1038/s41598-022-23400-9 (PMC9637207; doi:10.1038/s41598-022-23400-9)
Supplement: Supplementary file 1 — Supplementary Information 1. [file 41598_2022_23400_MOESM1_ESM.docx]

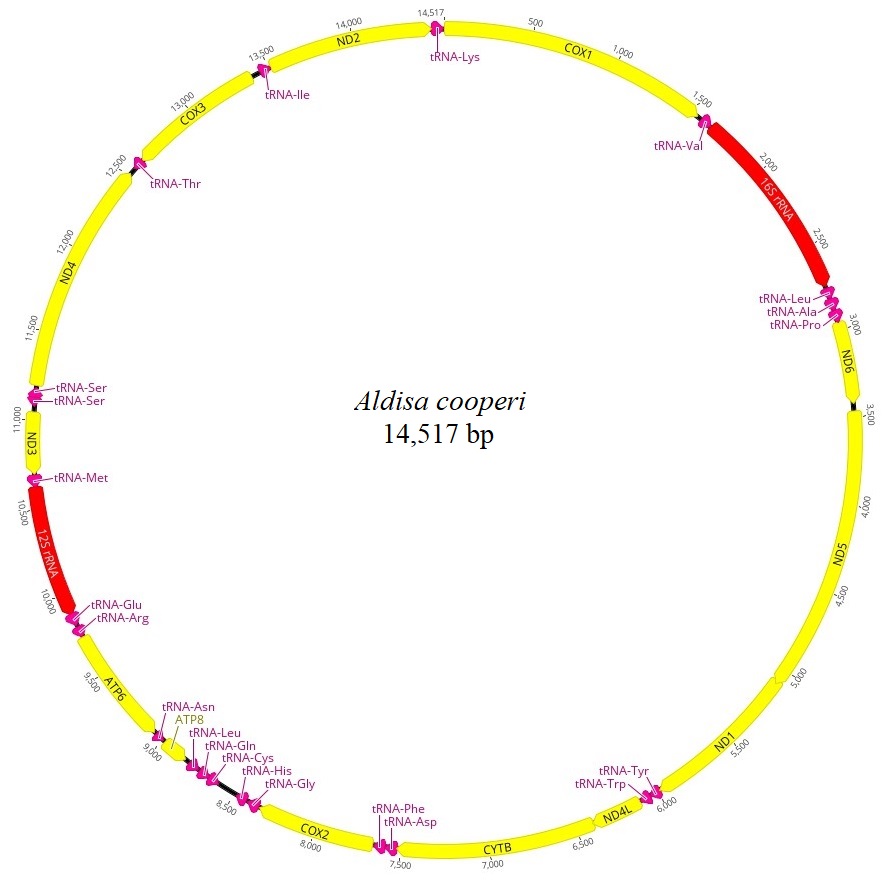


**Figure S1.** Gene map of compete mitogenome of *Aldisa cooperi*.


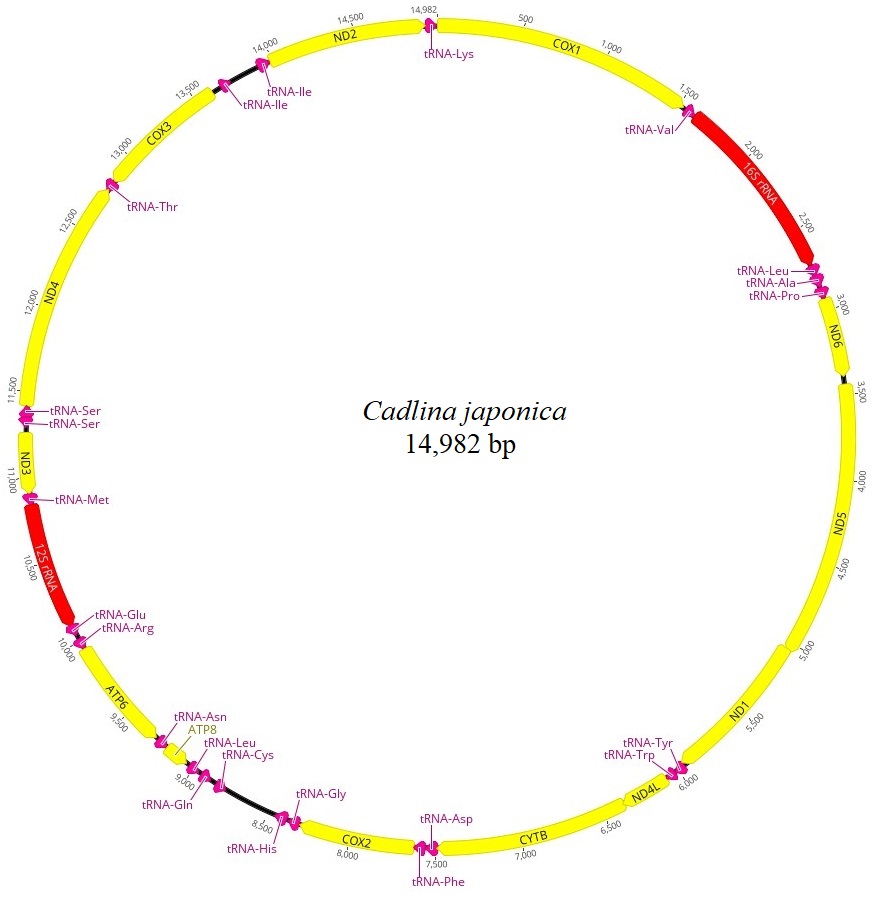


**Figure S2.** Gene map of compete mitogenome of *Cadlina japonica*.


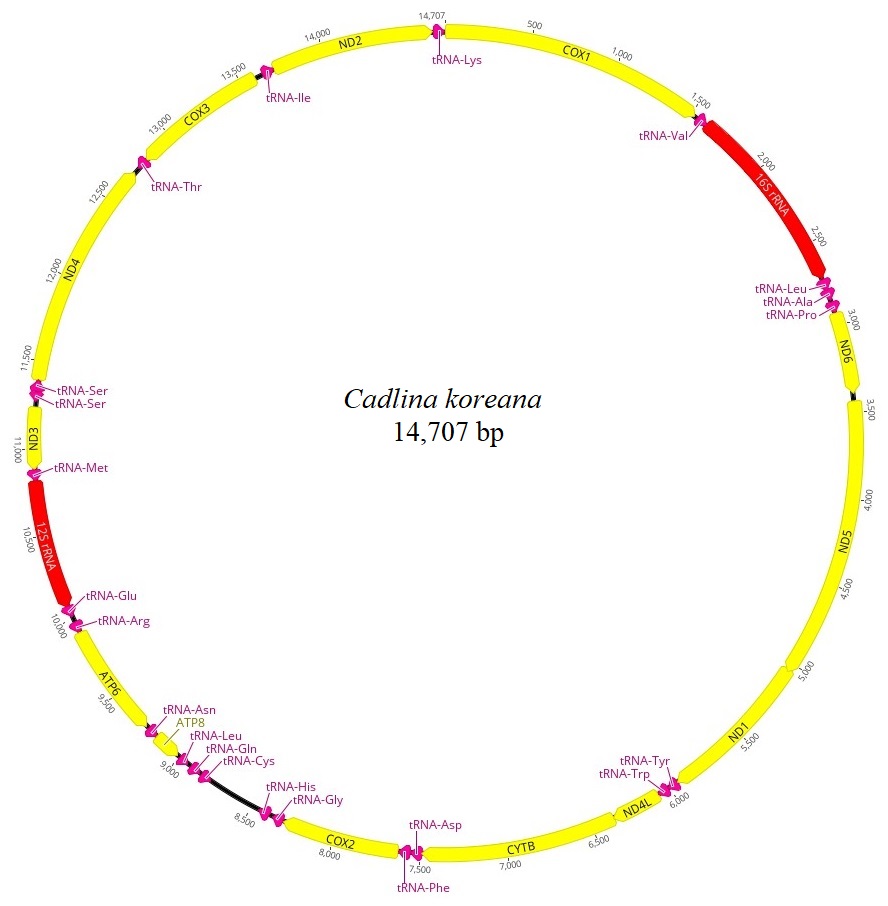


**Figure S3.** Gene map of compete mitogenome of *Cadlina koreana*.


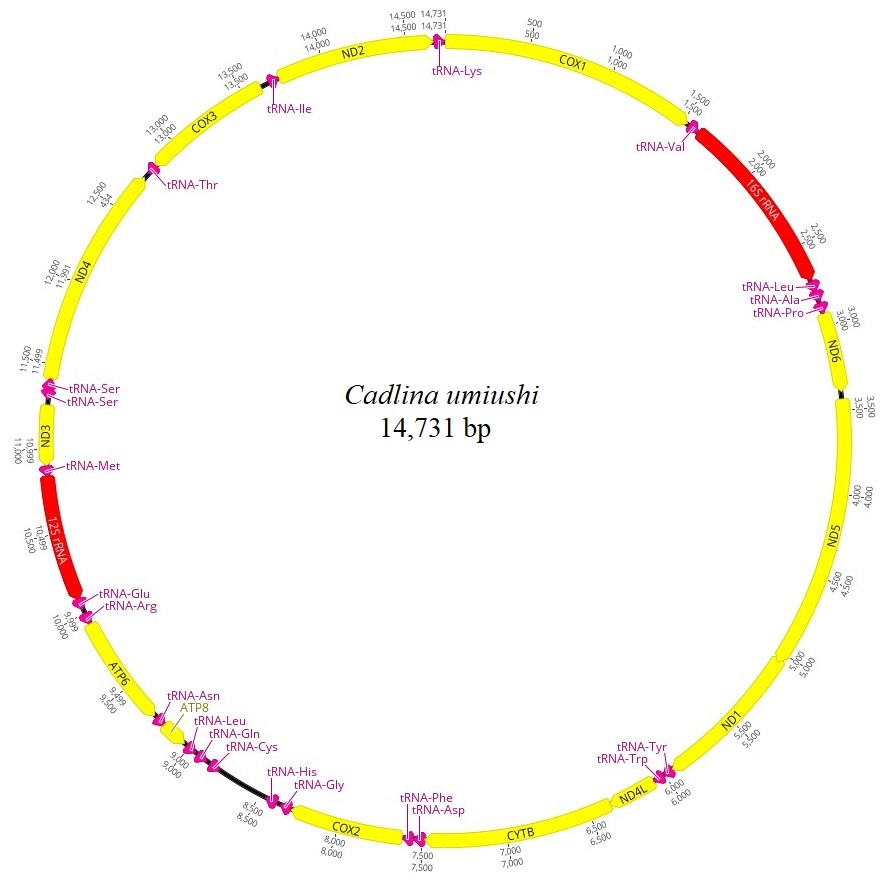


**Figure S4.** Gene map of compete mitogenome of *Cadlina umiushi*.


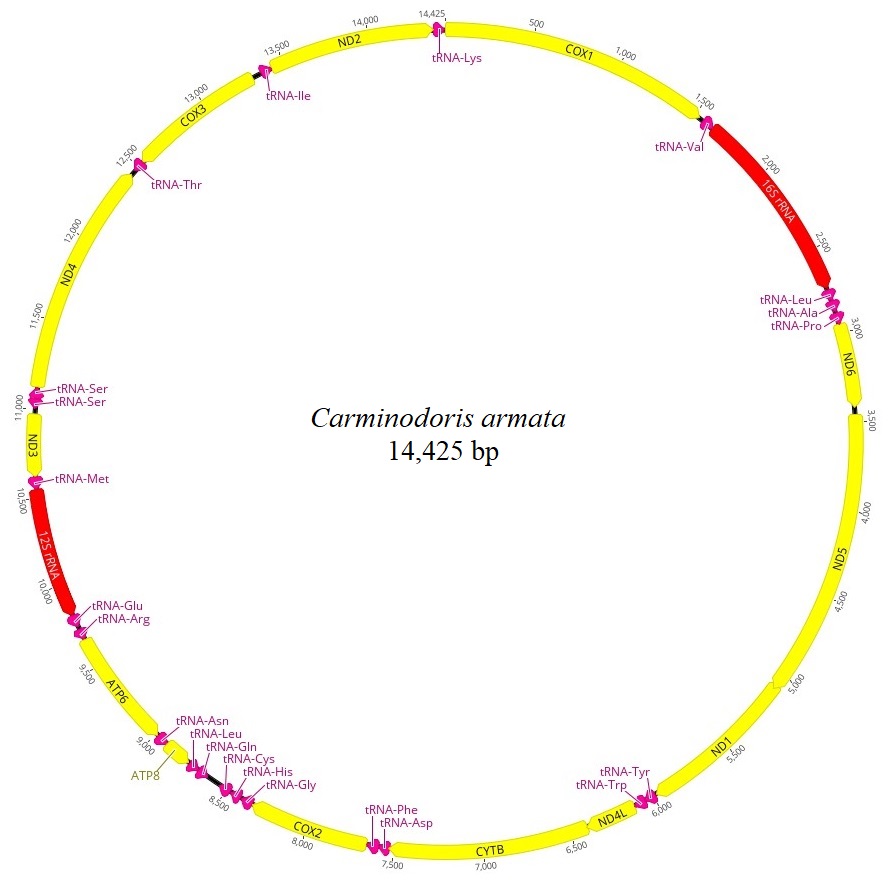


**Figure S5.** Gene map of compete mitogenome of *Carminodoris armata*.


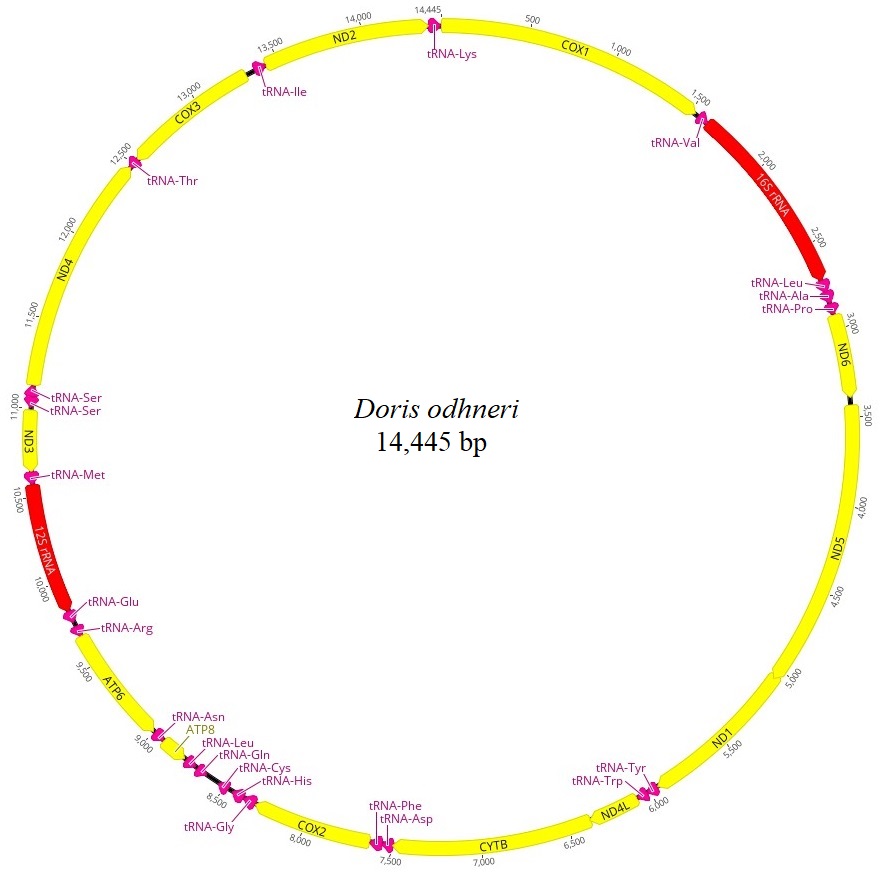


**Figure S6.** Gene map of compete mitogenome of *Doris odhneri*.


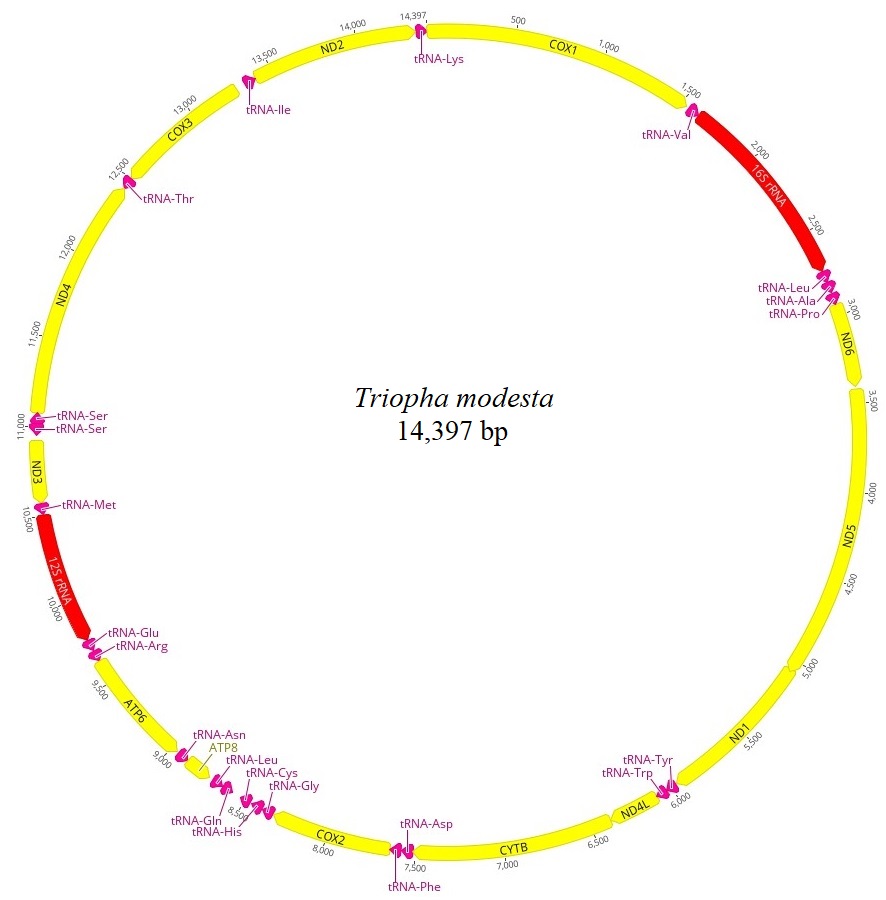


**Figure S7.** Gene map of compete mitogenome of *Triopha modesta*.


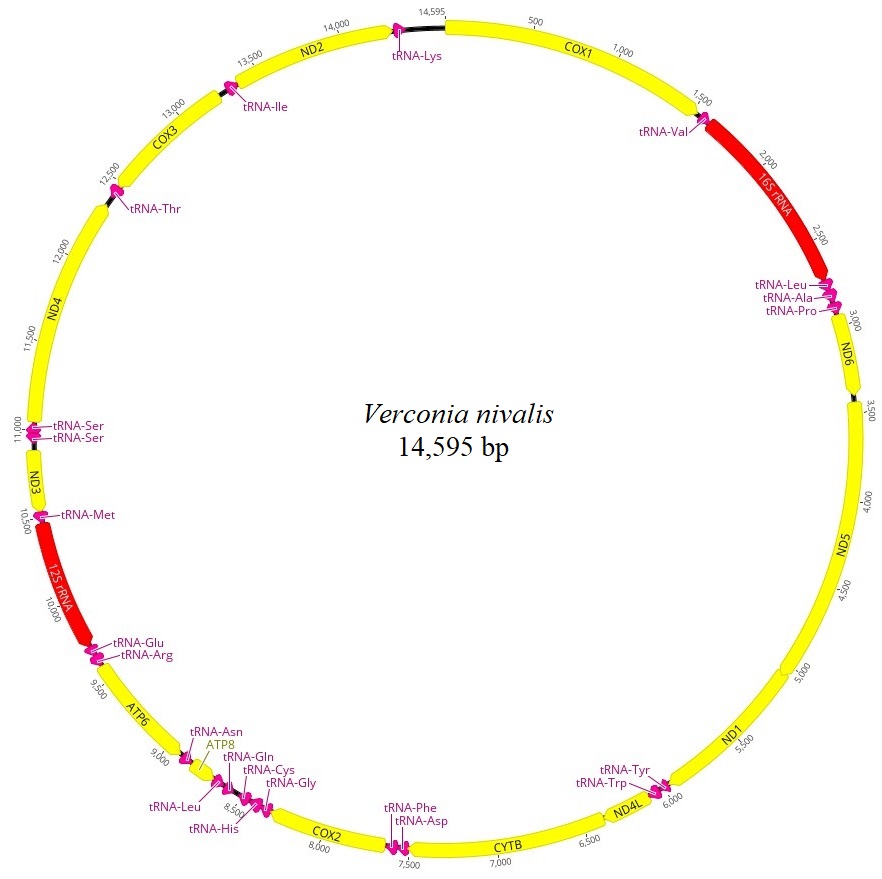


**Figure S8.** Gene map of compete mitogenome of *Verconia nivalis*.


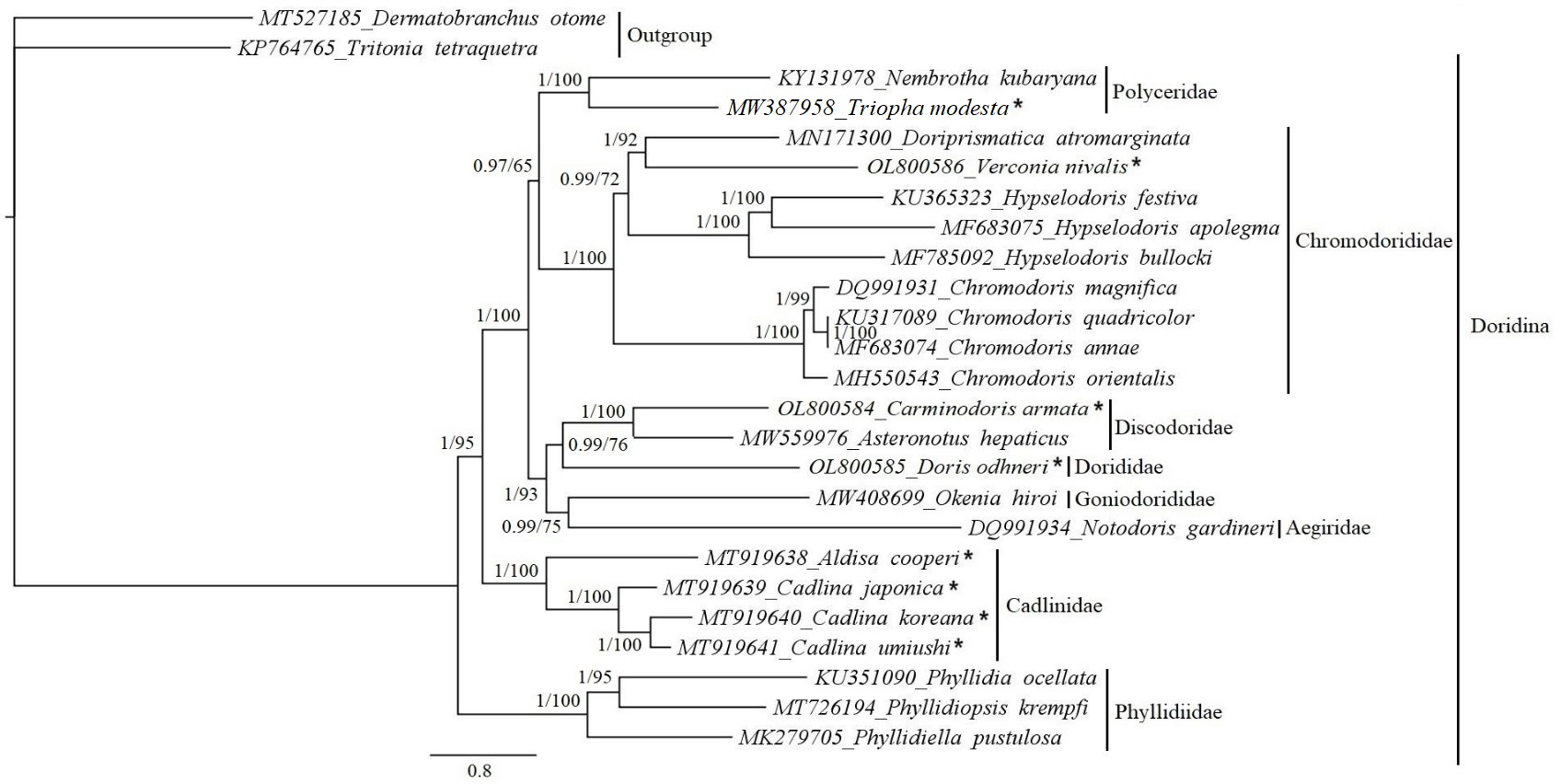


**Figure S9.** Phylogenetic tree showing the interfamily relationships of dorid nudibranchs based on the nucleotide sequences of 12 PCGs + 2 rRNAs + 22 tRNAs from mitogenomes (*nd4l* excluded). Sequences generated in this study are marked with stars. GenBank accession numbers are indicated next to species names. Gblocks was not used after sequence alignment. Posterior possibility values (left) and ultrafast bootstrap values (right) are shown at the nodes. Species of the suborder Cladobranchia were used as outgroup.


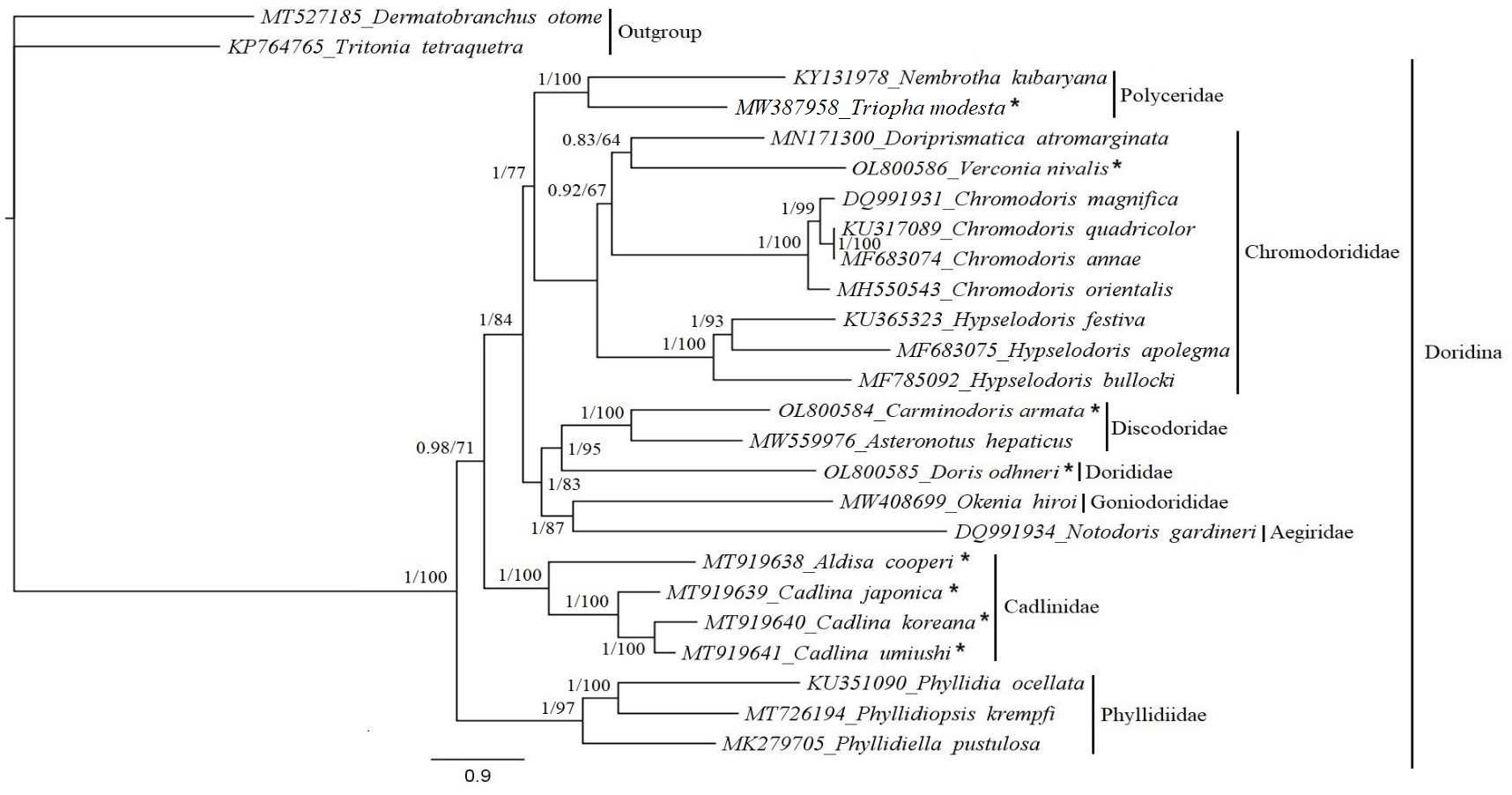


**Figure S10.** Phylogenetic tree showing the interfamily relationships of dorid nudibranchs based on the nucleotide sequences of 12 PCGs from mitogenomes (*nd4l* excluded). Sequences generated in this study are marked with stars. GenBank accession numbers are indicated next to species names. Gblocks was used after sequence alignment. Posterior possibility values (left) and ultrafast bootstrap values (right) are shown at the nodes. Species of the suborder Cladobranchia were used as outgroup.


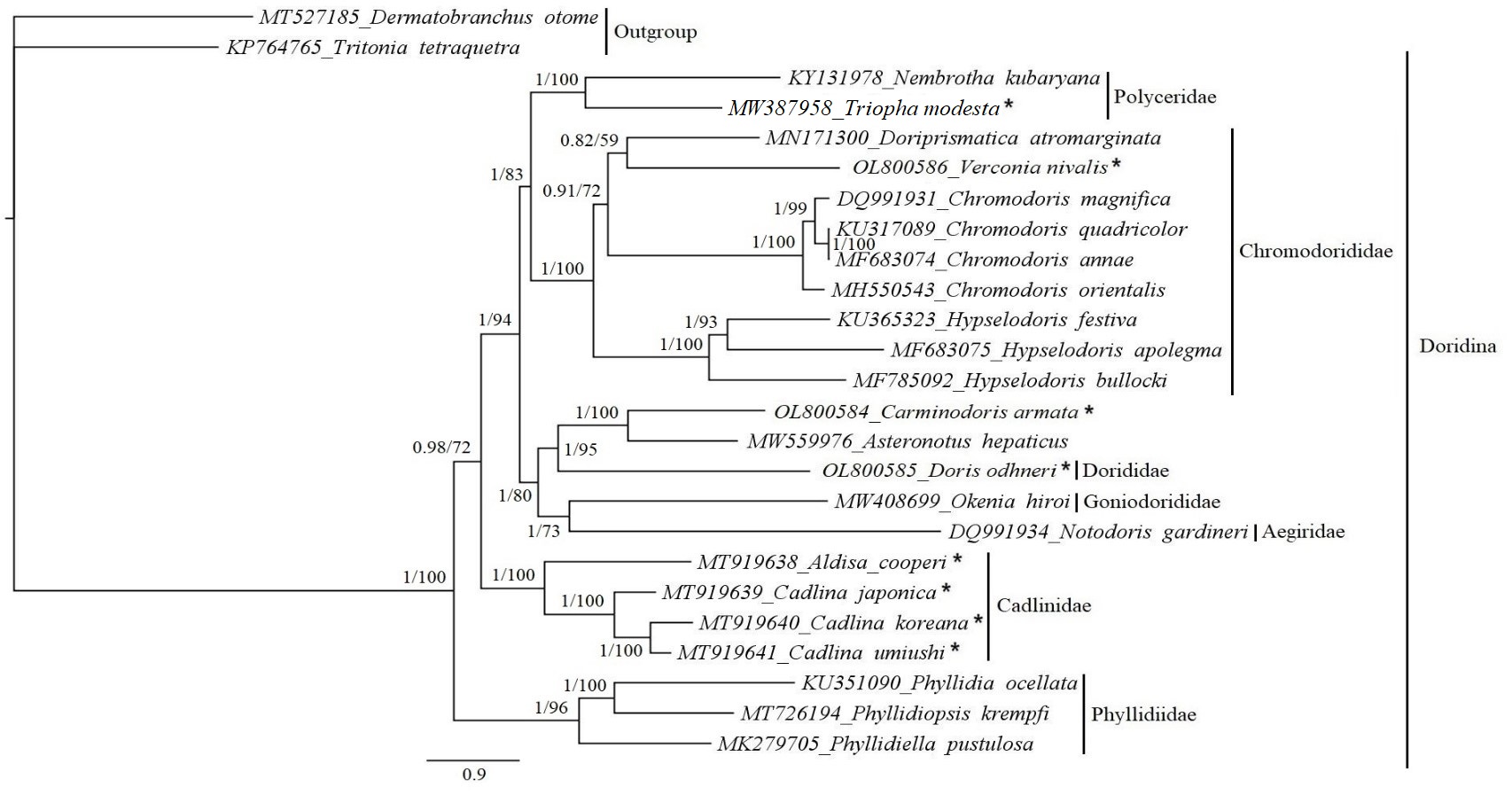


**Figure S11.** Phylogenetic tree showing the interfamily relationships of dorid nudibranchs based on the nucleotide sequences of 12 PCGs (*nd4l* excluded). Sequences generated in this study are marked with stars. GenBank accession numbers are indicated next to species names. Gblocks was not used after sequence alignment. Posterior possibility values (left) and ultrafast bootstrap values (right) are shown at the nodes. Species of the suborder Cladobranchia were used as outgroup.


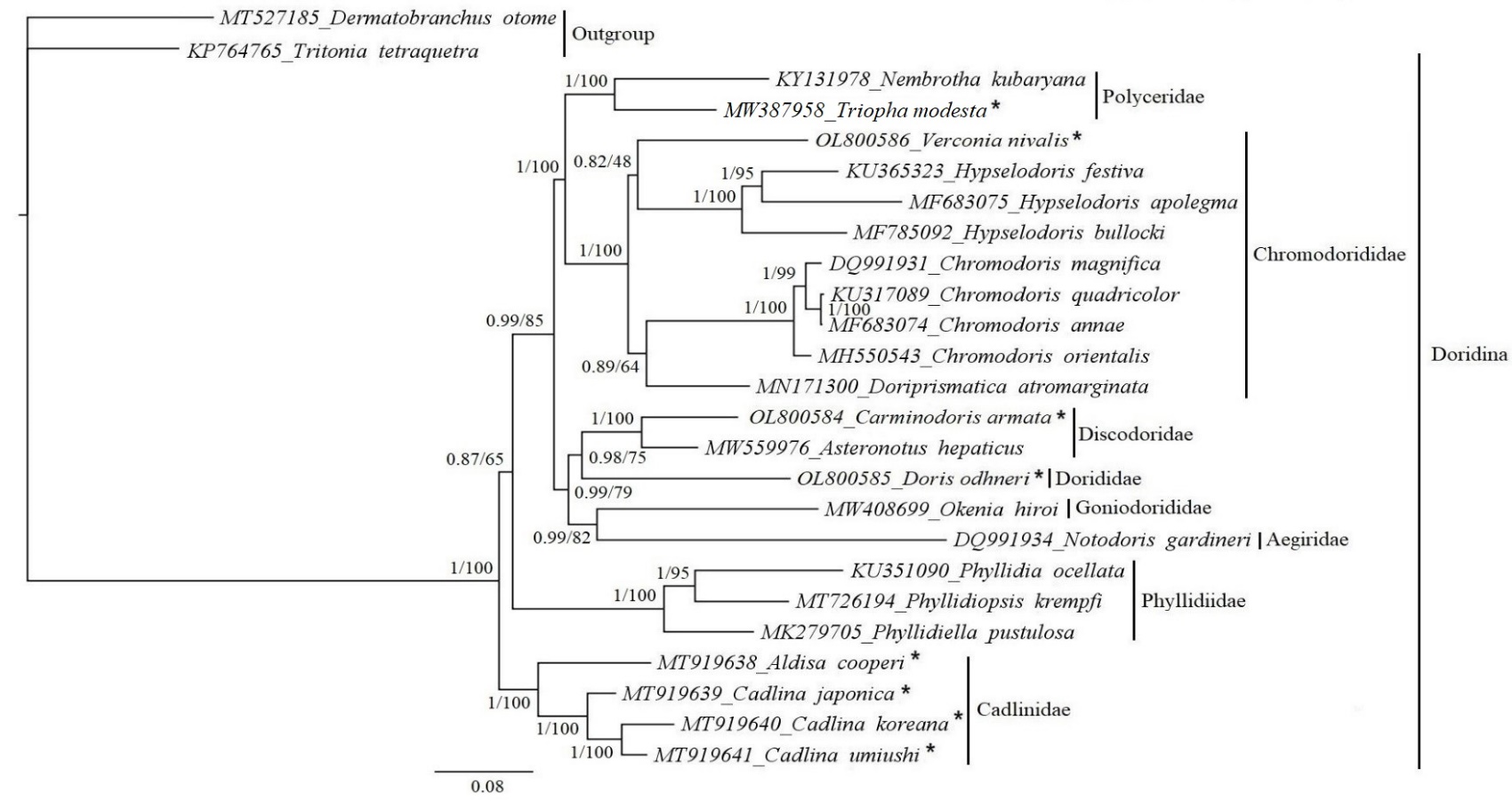


**Figure S12.** Phylogenetic tree showing the interfamily relationships of dorid nudibranchs based on 1st and 2nd codons of 12 PCGs (*nd4l* excluded). Sequences generated in this study are marked with stars. GenBank accession numbers are indicated next to species names. Gblocks was used after sequence alignment. Posterior possibility values (left) and ultrafast bootstrap values (right) are shown at the nodes. Species of the suborder Cladobranchia were used as outgroup.


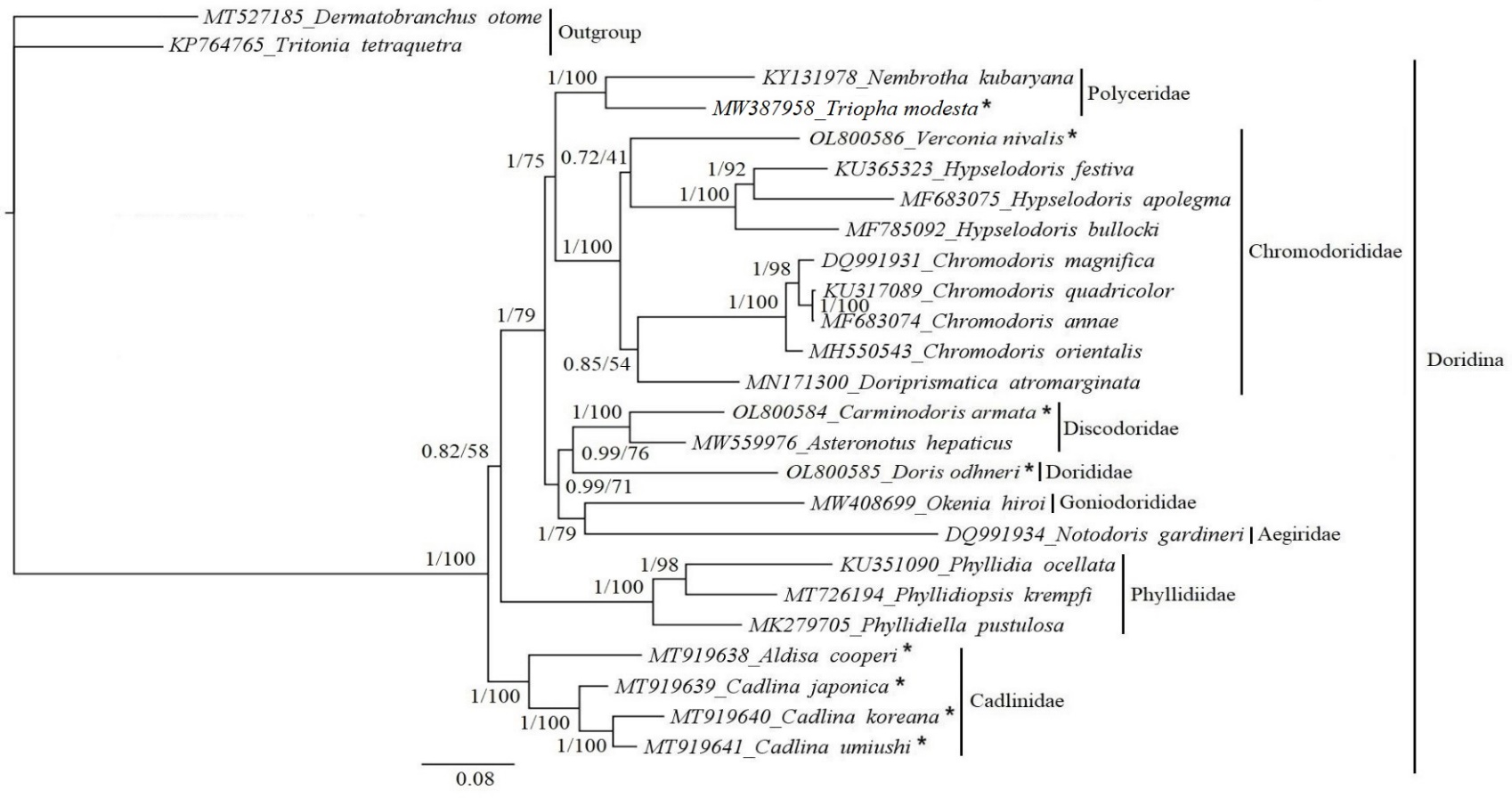


**Figure S13.** Phylogenetic tree showing the interfamily relationships of dorid nudibranchs based on 1st and 2nd codons of 12 PCGs (*nd4l* excluded). Sequences generated in this study are marked with stars. GenBank accession numbers are indicated next to species names. Gblocks was not used after sequence alignment. Posterior possibility values (left) and ultrafast bootstrap values (right) are shown at the nodes. Species of the suborder Cladobranchia were used as outgroup.


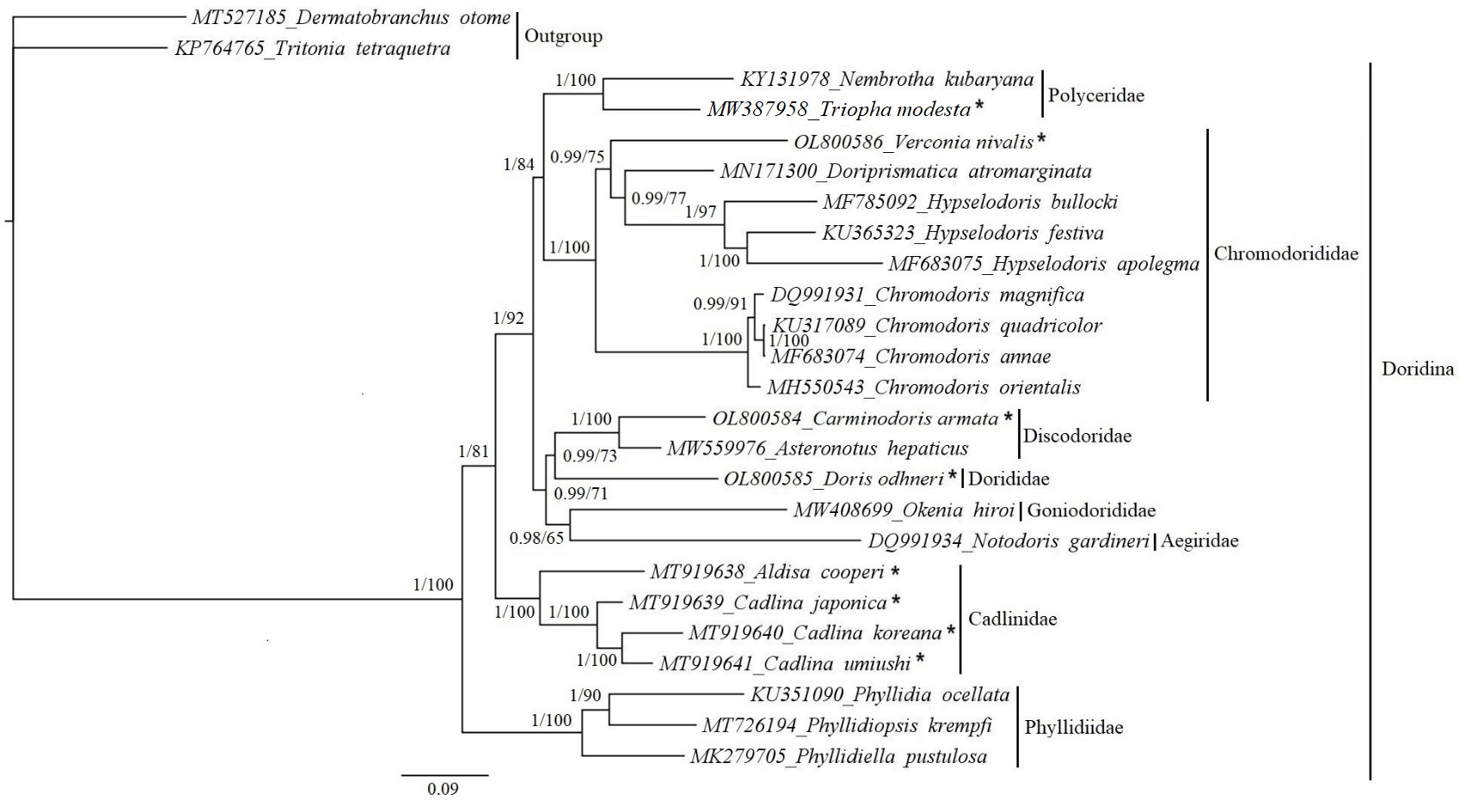


**Figure S14.** Phylogenetic tree showing the interfamily relationships of dorid nudibranchs based on the amino acid sequences of 12 PCGs (*nd4l* excluded). Sequences generated in this study are marked with stars. GenBank accession numbers are indicated next to species names. Gblocks was used after sequence alignment. Posterior possibility values (left) and ultrafast bootstrap values (right) are shown at the nodes. Species of the suborder Cladobranchia were used as outgroup.

**
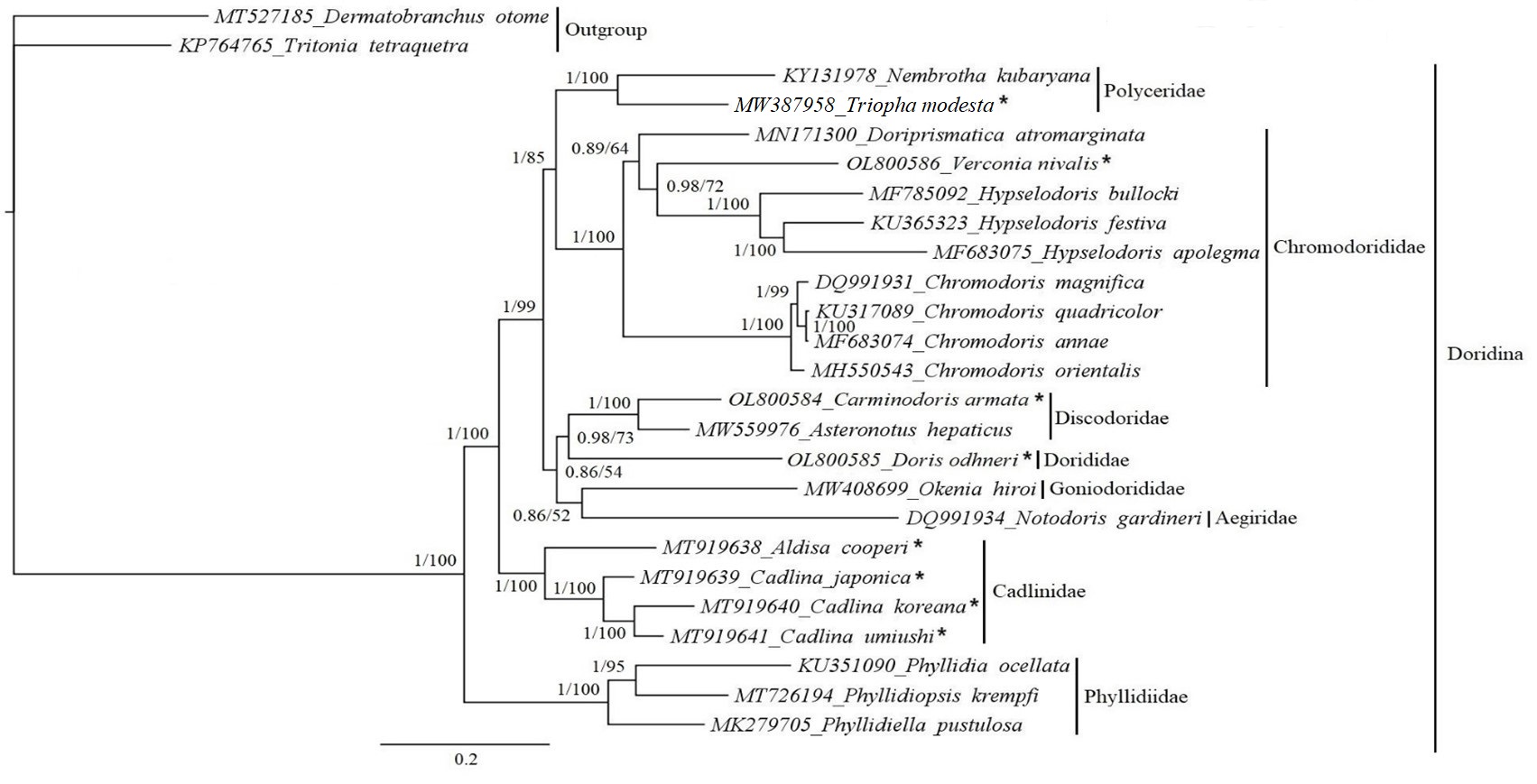
**

**Figure S15.** Phylogenetic tree showing the interfamily relationships of dorid nudibranchs based on the amino acid sequences of 12 PCGs (*nd4l* excluded). Sequences generated in this study are marked with stars. GenBank accession numbers are indicated next to species names. Gblocks was not used after sequence alignment. Posterior possibility values (left) and ultrafast bootstrap values (right) are shown at the nodes. Species of the suborder Cladobranchia were used as outgroup.

**
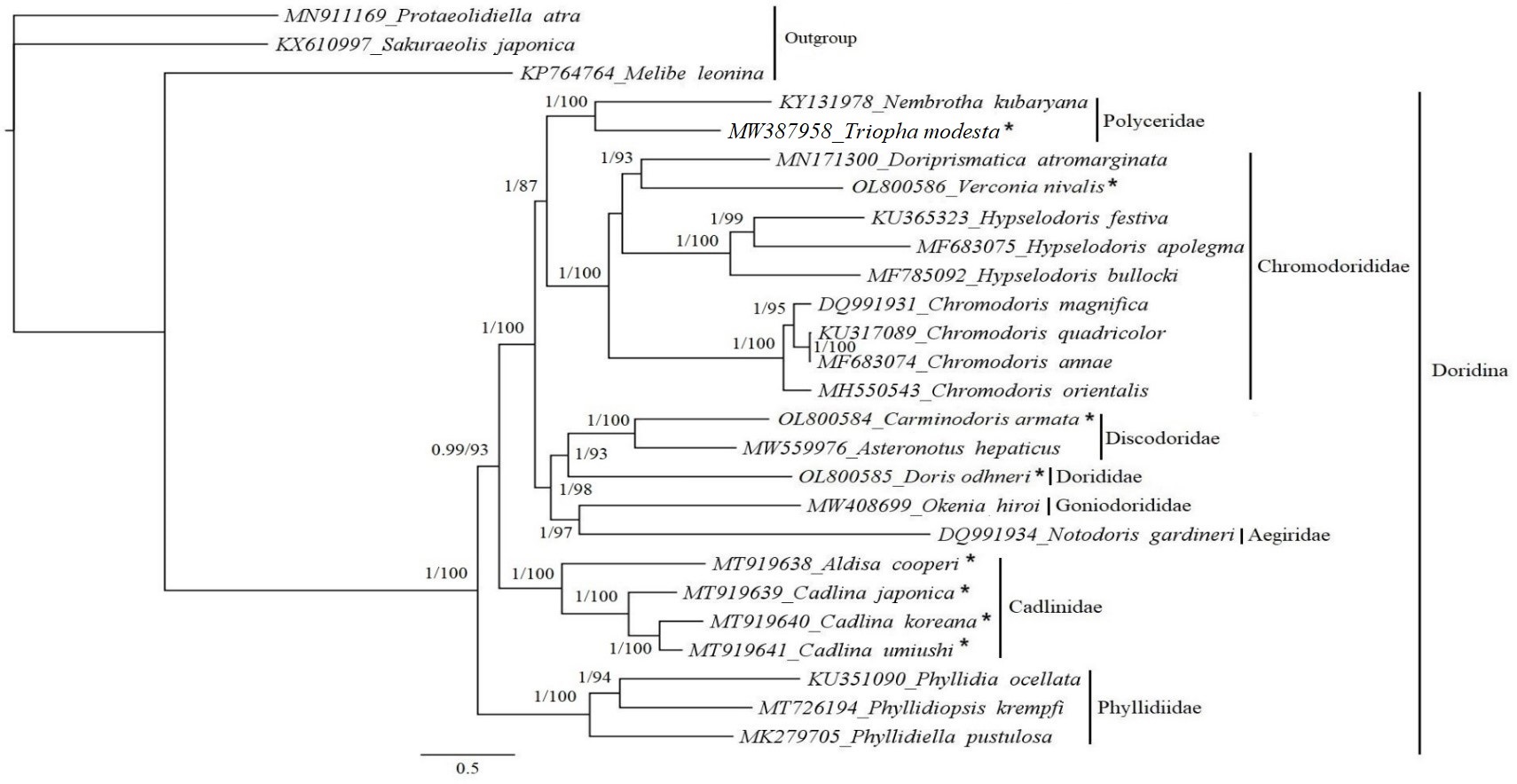
**

**Figure S16.** Phylogenetic tree showing the interfamily relationships of dorid nudibranchs based on the nucleotide sequences of 12 PCGs + 2 rRNAs + 22 tRNAs from mitogenomes (*nd4l* excluded). Sequences generated in this study are marked with stars. GenBank accession numbers are indicated next to species names. Gblocks was used after sequence alignment. Posterior possibility values (left) and ultrafast bootstrap values (right) are shown at the nodes. *Melibe leonina*, *Protaeolidiella atra* and *Sakuraeolis japonica* of the suborder Cladobranchia were used as outgroup.

**
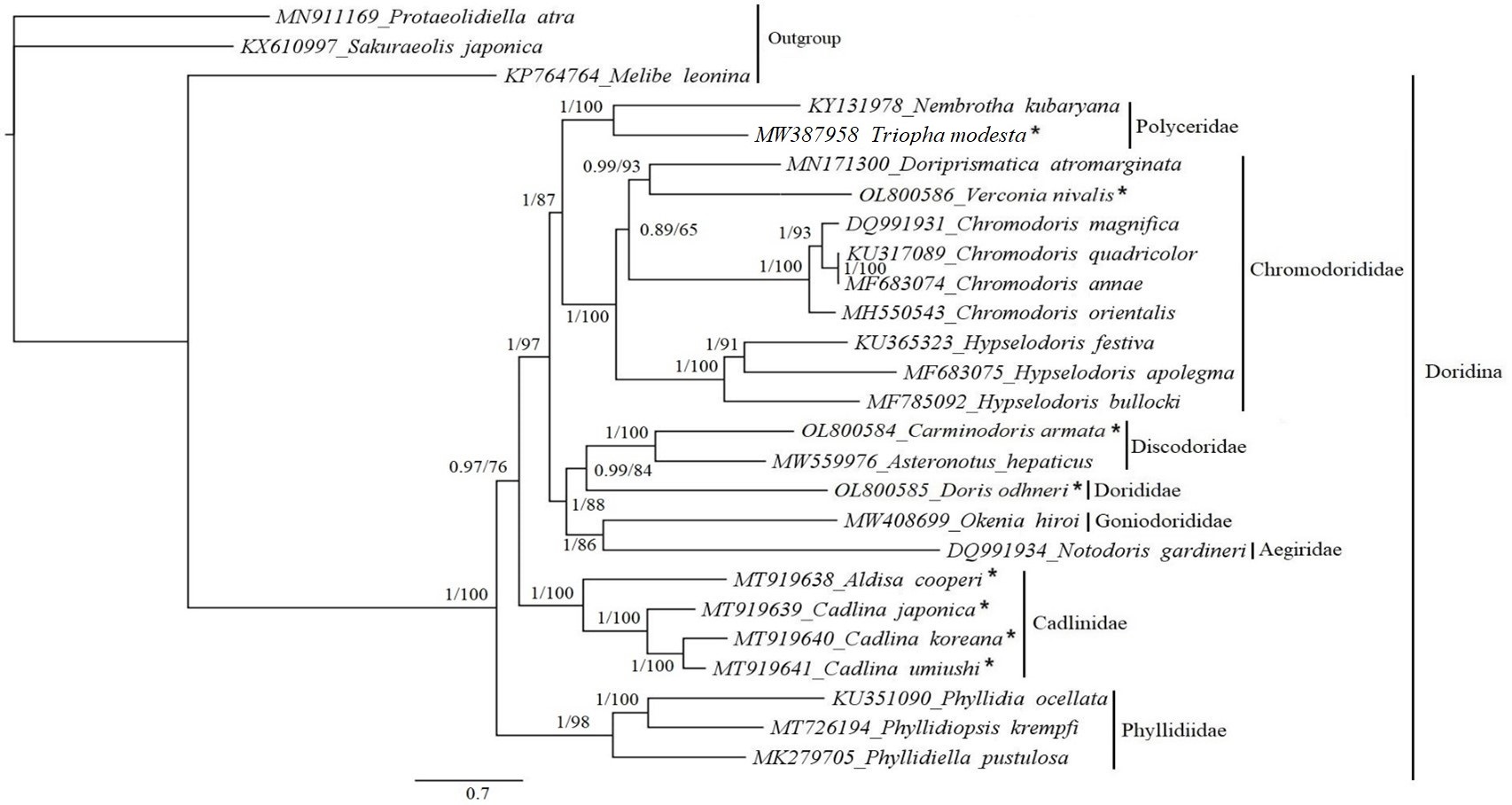
**

**Figure S17.** Phylogenetic tree showing the interfamily relationships of dorid nudibranchs based on the nucleotide sequences of 12 PCGs from mitogenomes (*nd4l* excluded). Sequences generated in this study are marked with stars. GenBank accession numbers are indicated next to species names. Gblocks was used after sequence alignment. Posterior possibility values (left) and ultrafast bootstrap values (right) are shown at the nodes. *Melibe leonina*, *Protaeolidiella atra* and *Sakuraeolis japonica* of the suborder Cladobranchia were used as outgroup.

**
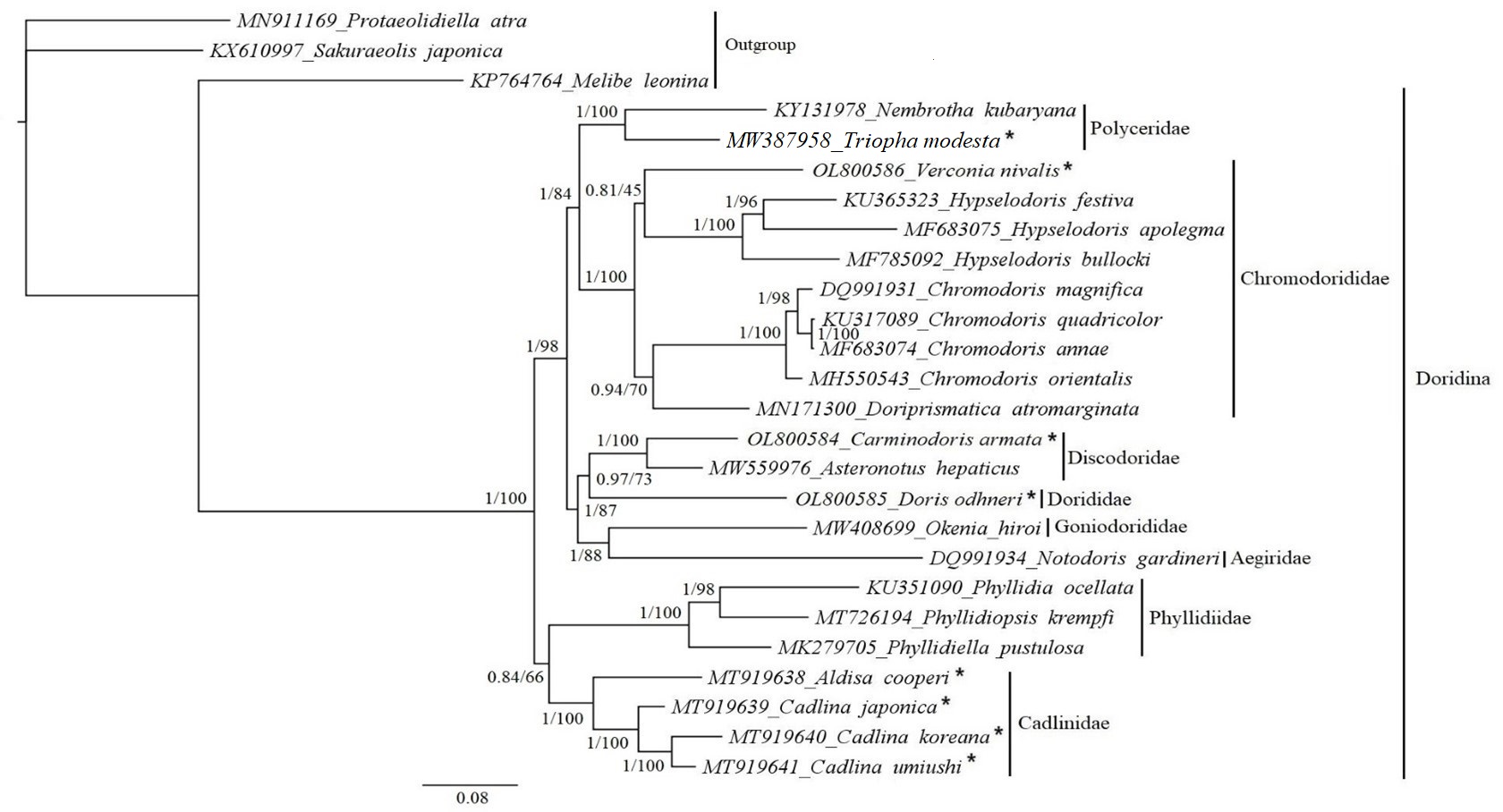
**

**Figure S18.** Phylogenetic tree showing the interfamily relationships of dorid nudibranchs based on 1st and 2nd codons of 12 PCGs (*nd4l* excluded). Sequences generated in this study are marked with stars. GenBank accession numbers are indicated next to species names. Gblocks was used after sequence alignment. Posterior possibility values (left) and ultrafast bootstrap values (right) are shown at the nodes. *Melibe leonina*, *Protaeolidiella atra* and *Sakuraeolis japonica* of the suborder Cladobranchia were used as outgroup.

**
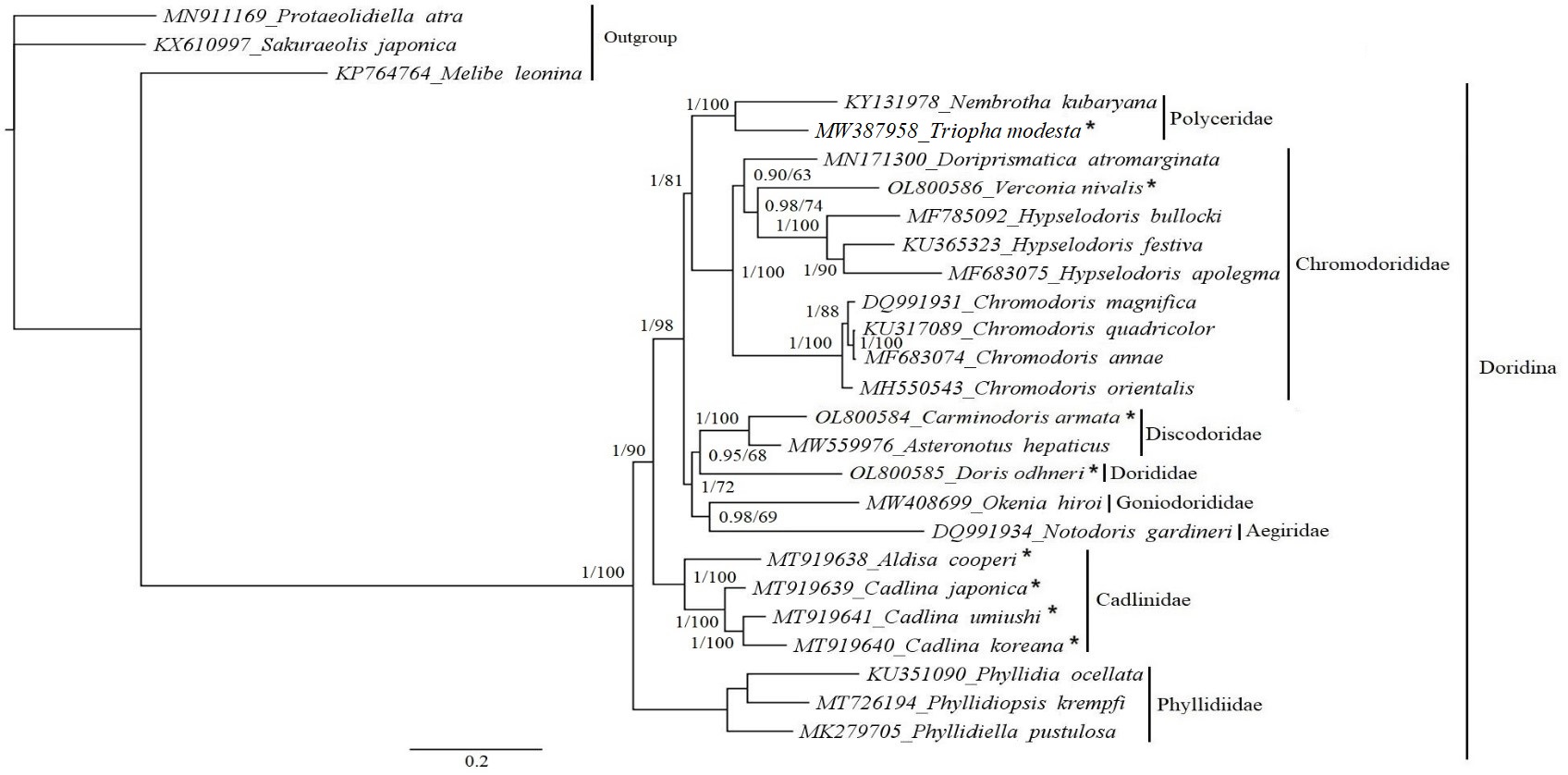
**

**Figure S19.** Phylogenetic tree showing the interfamily relationships of dorid nudibranchs based on the amino acid sequences of 12 PCGs (*nd4l* excluded). Sequences generated in this study are marked with stars. GenBank accession numbers are indicated next to species names. Gblocks was used after sequence alignment. Posterior possibility values (left) and ultrafast bootstrap values (right) are shown at the nodes. *Melibe leonina*, *Protaeolidiella atra* and *Sakuraeolis japonica* of the suborder Cladobranchia were used as outgroup.
