# Supplementary material for: Molecular phylogeny of selected dorid nudibranchs based on complete mitochondrial genome
Source: Sci Rep. 2022 Nov 5;12:18797. doi: 10.1038/s41598-022-23400-9 (PMC9637207; doi:10.1038/s41598-022-23400-9)
Supplement: Supplementary file 2 — Supplementary Information 2. [file 41598_2022_23400_MOESM2_ESM.docx]

**Table S1.** Gene structure of the *Aldisa cooperi* mitogenome. H or L indicates that the gene is encoded by the heavy or light strand, respectively.

| **Gene** | **Position** | | **Size** | **Codon** | | **Intergenic**  **nucleotide** | **Strand** |
| --- | --- | --- | --- | --- | --- | --- | --- |
|  | From | To |  | Start | Stop |  |  |
| *cox1* | 1 | 1,536 | 1,536 | ATG | TAG | 1 | H |
| tRNA^Val^ | 1,562 | 1,629 | 68 |  |  | 25 | H |
| 16S rRNA | 1,630 | 2,748 | 1,119 |  |  | 0 | H |
| tRNA^Leu^ | 2,758 | 2,823 | 66 |  |  | 9 | H |
| tRNA^Ala^ | 2,820 | 2,888 | 69 |  |  | -4 | H |
| tRNA^Pro^ | 2,892 | 2,957 | 66 |  |  | 3 | H |
| *nd6* | 2,958 | 3,425 | 468 | TTG | TAA | 0 | H |
| *nd5* | 3,469 | 5,091 | 1,623 | ATG | TAG | 43 | H |
| *nd1* | 5,072 | 5,983 | 912 | GTG | TAA | -20 | H |
| tRNA^Tyr^ | 5,983 | 6,044 | 62 |  |  | -1 | H |
| tRNA^Trp^ | 6,047 | 6,112 | 66 |  |  | 2 | H |
| *nd4l* | 6,113 | 6,406 | 294 | ATG | TAA | 0 | H |
| *cytb* | 6,399 | 7,526 | 1,128 | ATG | TAG | -8 | H |
| tRNA^Asp^ | 7,522 | 7,591 | 70 |  |  | -5 | H |
| tRNA^Phe^ | 7,593 | 7,658 | 66 |  |  | 1 | H |
| *cox2* | 7,660 | 8,334 | 675 | ATG | TAA | 1 | H |
| tRNA^Gly^ | 8,342 | 8,407 | 66 |  |  | 7 | H |
| tRNA^His^ | 8,418 | 8,481 | 64 |  |  | 10 | H |
| tRNA^Cys^ | 8,600 | 8,666 | 67 |  |  | 118 | L |
| tRNA^Gln^ | 8,666 | 8,730 | 65 |  |  | -1 | L |
| tRNA^Leu^ | 8,738 | 8,804 | 67 |  |  | 7 | L |
| *atp8* | 8,828 | 8,983 | 156 | ATG | TAA | 23 | L |
| tRNA^Asn^ | 8,984 | 9,050 | 67 |  |  | 0 | L |
| *atp6* | 9,066 | 9,734 | 669 | GTG | TAA | 15 | L |
| tRNA^Arg^ | 9,735 | 9,799 | 65 |  |  | 0 | L |
| tRNA^Glu^ | 9,809 | 9,877 | 69 |  |  | 9 | L |
| 12S rRNA | 9,878 | 10,623 | 746 |  |  | 0 | L |
| tRNA^Met^ | 10,624 | 10,690 | 67 |  |  | 0 | L |
| *nd3* | 10,694 | 11,047 | 354 | ATG | TAA | 3 | L |
| tRNA^Ser^ | 11,078 | 11,134 | 57 |  |  | 30 | L |
| tRNA^Ser^ | 11,140 | 11,199 | 60 |  |  | 5 | H |
| *nd4* | 11,200 | 12,519 | 1,320 | ATG | TAA | 0 | H |
| tRNA^Thr^ | 12,553 | 12,617 | 65 |  |  | 33 | L |
| *cox3* | 12,618 | 13,392 | 775 | ATG | T-- | 0 | L |
| tRNA^Ile^ | 13,446 | 13,513 | 68 |  |  | 53 | H |
| *nd2* | 13,514 | 14,449 | 936 | ATG | TAA | 0 | H |
| tRNA^Lys^ | 14,452 | 14,516 | 65 |  |  | 2 | H |

**Table S2.** Gene structure of the *Cadlina japonica* mitogenome. H or L indicates that the gene is encoded by the heavy or light strand, respectively.

| **Gene** | **Position** | | **Size** | **Codon** | | **Intergenic nucleotide** | **Strand** |
| --- | --- | --- | --- | --- | --- | --- | --- |
|  | From | To |  | Start | Stop |  |  |
| *cox1* | 1 | 1,530 | 1,530 | ATG | TAG | 1 | H |
| tRNA^Val^ | 1,545 | 1,613 | 69 |  |  | 14 | H |
| 16S rRNA | 1,614 | 2,718 | 1,105 |  |  | 0 | H |
| tRNA^Leu^ | 2,725 | 2,790 | 66 |  |  | 6 | H |
| tRNA^Ala^ | 2,787 | 2,854 | 68 |  |  | -4 | H |
| tRNA^Pro^ | 2,865 | 2,928 | 64 |  |  | 10 | H |
| *nd6* | 2,930 | 3,397 | 468 | TTG | TAA | 1 | H |
| *nd5* | 3,441 | 5,063 | 1,623 | ATA | TAG | 43 | H |
| *nd1* | 5,041 | 5,955 | 915 | TTG | TAA | -23 | H |
| tRNA^Tyr^ | 5,955 | 6,017 | 63 |  |  | -1 | H |
| tRNA^Trp^ | 6,020 | 6,082 | 63 |  |  | 2 | H |
| *nd4l* | 6,083 | 6,376 | 294 | GTG | TAA | 0 | H |
| *cytb* | 6,369 | 7,496 | 1,128 | ATG | TAA | -8 | H |
| tRNA^Asp^ | 7,492 | 7,559 | 68 |  |  | -5 | H |
| tRNA^Phe^ | 7,561 | 7,625 | 65 |  |  | 1 | H |
| *cox2* | 7,627 | 8,301 | 675 | GTG | TAA | 1 | H |
| tRNA^Gly^ | 8,308 | 8,372 | 65 |  |  | 6 | H |
| tRNA^His^ | 8,389 | 8,455 | 67 |  |  | 16 | H |
| tRNA^Cys^ | 8,780 | 8,845 | 66 |  |  | 324 | L |
| tRNA^Gln^ | 8,881 | 8,945 | 65 |  |  | 35 | L |
| tRNA^Leu^ | 8,964 | 9,027 | 64 |  |  | 18 | L |
| *atp8* | 9,050 | 9,202 | 153 | ATG | TAA | 22 | L |
| tRNA^Asn^ | 9,203 | 9,268 | 66 |  |  | 0 | L |
| *atp6* | 9,285 | 9,947 | 663 | ATG | TAA | 16 | L |
| tRNA^Arg^ | 9,948 | 10,013 | 66 |  |  | 0 | L |
| tRNA^Glu^ | 10,038 | 10,104 | 67 |  |  | 24 | L |
| 12S rRNA | 10,105 | 10,842 | 738 |  |  | 0 | L |
| tRNA^Met^ | 10,843 | 10,908 | 66 |  |  | 0 | L |
| *nd3* | 10,909 | 11,262 | 354 | GTG | TAA | 0 | L |
| tRNA^Ser^ | 11,292 | 11,349 | 58 |  |  | 29 | L |
| tRNA^Ser^ | 11,356 | 11,415 | 60 |  |  | 6 | H |
| *nd4* | 11,416 | 12,774 | 1,359 | ATG | TAG | 0 | H |
| tRNA^Thr^ | 12,769 | 12,834 | 66 |  |  | -6 | L |
| *cox3* | 12,835 | 13,609 | 775 | ATG | T-- | 0 | L |
| tRNA^Ile^ | 13,663 | 13,730 | 68 |  |  | 53 | H |
| tRNA^Ile^ | 13,912 | 13,979 | 68 |  |  | 181 | H |
| *nd2* | 13,980 | 14,915 | 936 | ATG | TAA | 0 | H |
| tRNA^Lys^ | 14,918 | 14,981 | 64 |  |  | 2 | H |

**Table S3.** Gene structure of the *Cadlina koreana* mitogenome. H or L indicates that the gene is encoded by the heavy or light strand, respectively.

| **Gene** | **Position** | | **Size** | **Codon** | | **Intergenic**  **nucleotide** | **Strand** |
| --- | --- | --- | --- | --- | --- | --- | --- |
|  | From | To |  | Start | Stop |  |  |
| *cox1* | 1 | 1,530 | 1,530 | ATG | TAA | 1 | H |
| tRNA^Val^ | 1,545 | 1,610 | 66 |  |  | 14 | H |
| 16S rRNA | 1,611 | 2,715 | 1,105 |  |  | 0 | H |
| tRNA^Leu^ | 2,722 | 2,787 | 66 |  |  | 6 | H |
| tRNA^Ala^ | 2,784 | 2,848 | 65 |  |  | -4 | H |
| tRNA^Pro^ | 2,859 | 2,922 | 64 |  |  | 10 | H |
| *nd6* | 2,924 | 3,391 | 468 | TTG | TAA | 1 | H |
| *nd5* | 3,435 | 5,057 | 1,623 | ATG | TAA | 43 | H |
| *nd1* | 5,038 | 5,949 | 912 | GTG | TAA | -20 | H |
| tRNA^Tyr^ | 5,949 | 6,010 | 62 |  |  | -1 | H |
| tRNA^Trp^ | 6,014 | 6,075 | 62 |  |  | 3 | H |
| *nd4l* | 6,076 | 6,369 | 294 | GTG | TAA | 0 | H |
| *cytb* | 6,362 | 7,489 | 1,128 | ATG | TAA | -8 | H |
| tRNA^Asp^ | 7,485 | 7,552 | 68 |  |  | -5 | H |
| tRNA^Phe^ | 7,554 | 7,619 | 66 |  |  | 1 | H |
| *cox2* | 7,621 | 8,295 | 675 | ATG | TAA | 1 | H |
| tRNA^Gly^ | 8,302 | 8,366 | 65 |  |  | 6 | H |
| tRNA^His^ | 8,389 | 8,453 | 65 |  |  | 22 | H |
| tRNA^Cys^ | 8,774 | 8,836 | 63 |  |  | 320 | L |
| tRNA^Gln^ | 8,850 | 8,914 | 65 |  |  | 13 | L |
| tRNA^Leu^ | 8,933 | 8,996 | 64 |  |  | 18 | L |
| *atp8* | 9,010 | 9,171 | 162 | ATG | TAA | 13 | L |
| tRNA^Asn^ | 9,172 | 9,237 | 66 |  |  | 0 | L |
| *atp6* | 9,251 | 9,913 | 663 |  |  | 13 | L |
| tRNA^Arg^ | 9,914 | 9,979 | 66 | ATG | TAA | 0 | L |
| tRNA^Glu^ | 10,008 | 10,074 | 67 |  |  | 28 | L |
| 12S rRNA | 10,075 | 10,814 | 740 |  |  | 0 | L |
| tRNA^Met^ | 10,815 | 10,880 | 66 |  |  | 0 | L |
| *nd3* | 10,880 | 11,233 | 354 | ATG | TAA | -1 | L |
| tRNA^Ser^ | 11,267 | 11,323 | 57 |  |  | 33 | L |
| tRNA^Ser^ | 11,330 | 11,389 | 60 |  |  | 6 | H |
| *nd4* | 11,390 | 12,712 | 1,323 | GTG | TAA | 0 | H |
| tRNA^Thr^ | 12,743 | 12,807 | 65 |  |  | 30 | L |
| *cox3* | 12,808 | 13,582 | 775 | ATG | T-- | 0 | L |
| tRNA^Ile^ | 13,636 | 13,704 | 69 |  |  | 53 | H |
| *nd2* | 13,705 | 14,640 | 936 | ATG | TAA | 0 | H |
| tRNA^Lys^ | 14,643 | 14,706 | 64 |  |  | 2 | H |

**Table S4.** Gene structure of the *Cadlina umiushi* mitogenome. H or L indicates that the gene is encoded by the heavy or light strand, respectively.

| **Gene** | **Position** | | **Size** | **Codon** | | **Intergenic**  **nucleotide** | **Strand** |
| --- | --- | --- | --- | --- | --- | --- | --- |
|  | From | To |  | Start | Stop |  |  |
| *cox1* | 1 | 1,530 | 1,530 | ATG | TAG | 1 | H |
| tRNA^Val^ | 1,545 | 1,612 | 68 |  |  | 14 | H |
| 16S rRNA | 1,613 | 2,714 | 1,102 |  |  | 0 | H |
| tRNA^Leu^ | 2,721 | 2,786 | 66 |  |  | 6 | H |
| tRNA^Ala^ | 2,783 | 2,847 | 65 |  |  | -4 | H |
| tRNA^Pro^ | 2,858 | 2,921 | 64 |  |  | 10 | H |
| *nd6* | 2,923 | 3,390 | 468 | TTG | TAA | 1 | H |
| *nd5* | 3,434 | 5,056 | 1,623 | ATA | TAG | 43 | H |
| *nd1* | 5,037 | 5,948 | 912 | GTG | TAA | -20 | H |
| tRNA^Tyr^ | 5,948 | 6,009 | 62 |  |  | -1 | H |
| tRNA^Trp^ | 6,013 | 6,075 | 63 |  |  | 3 | H |
| *nd4l* | 6,076 | 6,369 | 294 | GTG | TAA | 0 | H |
| *cytb* | 6,362 | 7,489 | 1,128 | ATG | TAA | -8 | H |
| tRNA^Asp^ | 7,485 | 7,553 | 69 |  |  | -5 | H |
| tRNA^Phe^ | 7,555 | 7,619 | 65 |  |  | 1 | H |
| *cox2* | 7,621 | 8,295 | 675 | GTG | TAA | 1 | H |
| tRNA^Gly^ | 8,302 | 8,366 | 65 |  |  | 6 | H |
| tRNA^His^ | 8,389 | 8,454 | 66 |  |  | 22 | H |
| tRNA^Cys^ | 8,776 | 8,841 | 66 |  |  | 321 | L |
| tRNA^Gln^ | 8,871 | 8,935 | 65 |  |  | 29 | L |
| tRNA^Leu^ | 8,952 | 9,015 | 64 |  |  | 16 | L |
| *atp8* | 9,035 | 9,190 | 156 | ATG | TAA | 19 | L |
| tRNA^Asn^ | 9,191 | 9,256 | 66 |  |  | 0 | L |
| *atp6* | 9,273 | 9,935 | 663 | ATG | TAA | 16 | L |
| tRNA^Arg^ | 9,936 | 10,002 | 67 |  |  | 0 | L |
| tRNA^Glu^ | 10,032 | 10,098 | 67 |  |  | 29 | L |
| 12S rRNA | 10,099 | 10,838 | 740 |  |  | 0 | L |
| tRNA^Met^ | 10,839 | 10,904 | 66 |  |  | 0 | L |
| *nd3* | 10,904 | 11,257 | 354 | ATG | TAA | -1 | L |
| tRNA^Ser^ | 11,293 | 11,349 | 57 |  |  | 35 | L |
| tRNA^Ser^ | 11,356 | 11,415 | 60 |  |  | 6 | H |
| *nd4* | 11,416 | 12,738 | 1,323 | GTG | TAA | 0 | H |
| tRNA^Thr^ | 12,769 | 12,831 | 63 |  |  | 30 | L |
| *cox3* | 12,832 | 13,606 | 775 | ATG | T-- | 0 | L |
| tRNA^Ile^ | 13,660 | 13,728 | 69 |  |  | 53 | H |
| *nd2* | 13,729 | 14,664 | 936 | ATG | TAA | 0 | H |
| tRNA^Lys^ | 14,667 | 14,730 | 64 |  |  | 2 | H |

**Table S5.** Gene structure of the *Carminodoris armata* mitogenome. H or L indicates that the gene is encoded by the heavy or light strand, respectively.

| **Gene** | **Position** | | **Size** | **Codon** | | **Intergenic**  **nucleotide** | **Strand** |
| --- | --- | --- | --- | --- | --- | --- | --- |
|  | From | To |  | Start | Stop |  |  |
| *cox1* | 1 | 1,533 | 1,533 | ATG | TAA | 0 | H |
| tRNA^Val^ | 1,561 | 1,627 | 67 |  |  | 27 | H |
| 16S rRNA | 1,628 | 2,736 | 1,109 |  |  | 0 | H |
| tRNA^Leu^ | 2,745 | 2,810 | 66 |  |  | 8 | H |
| tRNA^Ala^ | 2,810 | 2,874 | 65 |  |  | -1 | H |
| tRNA^Pro^ | 2,883 | 2,946 | 64 |  |  | 8 | H |
| *nd6* | 2,948 | 3,415 | 468 | TTG | TAA | 1 | H |
| *nd5* | 3,459 | 5,084 | 1,626 | ATA | TAG | 43 | H |
| *nd1* | 5,065 | 5,976 | 912 | GTG | TAA | -20 | H |
| tRNA^Tyr^ | 5,984 | 6,049 | 66 |  |  | 7 | H |
| tRNA^Trp^ | 6,047 | 6,112 | 66 |  |  | -3 | H |
| *nd4l* | 6,113 | 6,406 | 294 | ATA | TAA | 0 | H |
| *cytb* | 6,399 | 7,526 | 1,128 | ATG | TAA | -8 | H |
| tRNA^Asp^ | 7,522 | 7,589 | 68 |  |  | -5 | H |
| tRNA^Phe^ | 7,590 | 7,656 | 67 |  |  | 0 | H |
| *cox2* | 7,657 | 8,331 | 675 | GTG | TAA | 0 | H |
| tRNA^Gly^ | 8,336 | 8,401 | 66 |  |  | 4 | H |
| tRNA^His^ | 8,408 | 8,473 | 66 |  |  | 6 | H |
| tRNA^Cys^ | 8,477 | 8,543 | 67 |  |  | 3 | H |
| tRNA^Gln^ | 8,625 | 8,686 | 62 |  |  | 81 | L |
| tRNA^Leu^ | 8,688 | 8,752 | 65 |  |  | 1 | L |
| *atp8* | 8,761 | 8,916 | 156 | ATG | TAA | 8 | L |
| tRNA^Asn^ | 8,917 | 8,984 | 68 |  |  | 0 | L |
| *atp6* | 8,993 | 9,661 | 669 | ATG | TAA | 8 | L |
| tRNA^Arg^ | 9,662 | 9,726 | 65 |  |  | 0 | L |
| tRNA^Glu^ | 9,746 | 9,813 | 68 |  |  | 19 | L |
| 12S rRNA | 9,814 | 10,549 | 736 |  |  | 0 | L |
| tRNA^Met^ | 10,550 | 10,615 | 66 |  |  | 0 | L |
| *nd3* | 10,617 | 10,970 | 354 | ATG | TAA | 1 | L |
| tRNA^Ser^ | 11,000 | 11,058 | 59 |  |  | 29 | L |
| tRNA^Ser^ | 11,063 | 11,122 | 60 |  |  | 4 | H |
| *nd4* | 11,123 | 12,437 | 1,315 | GTG | T-- | 0 | H |
| tRNA^Thr^ | 12,469 | 12,532 | 64 |  |  | 31 | L |
| *cox3* | 12,533 | 13,307 | 775 | ATG | TAG | 0 | L |
| tRNA^Ile^ | 13,362 | 13,427 | 66 |  |  | 54 | H |
| *nd2* | 13,428 | 14,363 | 936 | ATG | TAG | 0 | H |
| tRNA^Lys^ | 14,363 | 14,425 | 63 |  |  | -1 | H |

**Table S6.** Gene structure of the *Doris odhneri* mitogenome. H or L indicates that the gene is encoded by the heavy or light strand, respectively.

| **Gene** | **Position** | | **Size** | **Codon** | | **Intergenic**  **nucleotide** | **Strand** |
| --- | --- | --- | --- | --- | --- | --- | --- |
|  | From | To |  | Start | Stop |  |  |
| *cox1* | 1 | 1,533 | 1,533 | ATG | TAG | 0 | H |
| tRNA^Val^ | 1,553 | 1,618 | 66 |  |  | 19 | H |
| 16S rRNA | 1,619 | 2,718 | 1,100 |  |  | 0 | H |
| tRNA^Leu^ | 2,719 | 2,785 | 67 |  |  | 0 | H |
| tRNA^Ala^ | 2,782 | 2,849 | 68 |  |  | -4 | H |
| tRNA^Pro^ | 2,857 | 2,919 | 63 |  |  | 7 | H |
| *nd6* | 2,921 | 3,391 | 471 | TTG | TAA | 1 | H |
| *nd5* | 3,435 | 5,057 | 1,623 | ATA | TAG | 43 | H |
| *nd1* | 5,038 | 5,949 | 912 | GTG | TAA | -20 | H |
| tRNA^Tyr^ | 5,952 | 6,014 | 63 |  |  | 2 | H |
| tRNA^Trp^ | 6,016 | 6,080 | 65 |  |  | 1 | H |
| *nd4l* | 6,081 | 6,374 | 294 | ATG | TAA | 0 | H |
| *cytb* | 6,367 | 7,494 | 1,128 | ATG | TAA | -8 | H |
| tRNA^Asp^ | 7,490 | 7,556 | 67 |  |  | -5 | H |
| tRNA^Phe^ | 7,556 | 7,621 | 66 |  |  | -1 | H |
| *cox2* | 7,622 | 8,296 | 675 | ATG | TAA | 0 | H |
| tRNA^Gly^ | 8,304 | 8,369 | 66 |  |  | 7 | H |
| tRNA^His^ | 8,380 | 8,448 | 69 |  |  | 10 | H |
| tRNA^Cys^ | 8,472 | 8,538 | 67 |  |  | 23 | H |
| tRNA^Gln^ | 8,617 | 8,680 | 64 |  |  | 78 | L |
| tRNA^Leu^ | 8,696 | 8,761 | 66 |  |  | 15 | L |
| *atp8* | 8,770 | 8,925 | 156 | ATG | TAG | 8 | L |
| tRNA^Asn^ | 8,926 | 8,993 | 68 |  |  | 0 | L |
| *atp6* | 9,003 | 9,671 | 669 | ATG | TAG | 9 | L |
| tRNA^Arg^ | 9,672 | 9,736 | 65 |  |  | 0 | L |
| tRNA^Glu^ | 9,760 | 9,825 | 66 |  |  | 23 | L |
| 12S rRNA | 9,826 | 10,569 | 744 |  |  | 0 | L |
| tRNA^Met^ | 10,570 | 10,636 | 67 |  |  | 0 | L |
| *nd3* | 10,637 | 10,988 | 352 | ATG | T-- | 0 | L |
| tRNA^Ser^ | 10,999 | 11,058 | 60 |  |  | 10 | L |
| tRNA^Ser^ | 11,063 | 11,123 | 61 |  |  | 4 | H |
| *nd4* | 11,124 | 12,479 | 1,356 | ATG | TAG | 0 | H |
| tRNA^Thr^ | 12,474 | 12,537 | 64 |  |  | -6 | L |
| *cox3* | 12,538 | 13,312 | 775 | ATG | T-- | 0 | L |
| tRNA^Ile^ | 13,367 | 13,431 | 65 |  |  | 54 | H |
| *nd2* | 13,433 | 14,377 | 945 | ATG | TAA | 1 | H |
| tRNA^Lys^ | 14,379 | 14,445 | 67 |  |  | 1 | H |

**Table S7.** Gene structure of the *Triopha modesta* mitogenome. H or L indicates that the gene is encoded by the heavy or light strand, respectively.

| **Gene** | **Position** | | **Size** | **Codon** | | **Intergenic**  **nucleotide** | **Strand** |
| --- | --- | --- | --- | --- | --- | --- | --- |
|  | From | To |  | Start | Stop |  |  |
| *cox1* | 1 | 1,533 | 1,533 | ATG | TAA | 0 | H |
| tRNA^Val^ | 1,550 | 1,616 | 67 |  |  | 16 | H |
| 16S rRNA | 1,617 | 2,736 | 1,120 |  |  | 0 | H |
| tRNA^Leu^ | 2,738 | 2,804 | 67 |  |  | 1 | H |
| tRNA^Ala^ | 2,801 | 2,867 | 67 |  |  | -4 | H |
| tRNA^Pro^ | 2,872 | 2,935 | 64 |  |  | 4 | H |
| *nd6* | 2,937 | 3,407 | 471 | TTG | TAA | 1 | H |
| *nd5* | 3,424 | 5,073 | 1,650 | GTG | TAG | 16 | H |
| *nd1* | 5,054 | 5,965 | 912 | GTG | TAA | -20 | H |
| tRNA^Tyr^ | 5,970 | 6,031 | 62 |  |  | 4 | H |
| tRNA^Trp^ | 6,034 | 6,098 | 65 |  |  | 2 | H |
| *nd4l* | 6,099 | 6,386 | 288 | ATG | TAA | 0 | H |
| *cytb* | 6,387 | 7,514 | 1,128 | ATG | TAA | 0 | H |
| tRNA^Asp^ | 7,510 | 7,576 | 67 |  |  | -5 | H |
| tRNA^Phe^ | 7,577 | 7,642 | 66 |  |  | 0 | H |
| *cox2* | 7,644 | 8,318 | 675 | ATG | TAA | 1 | H |
| tRNA^Gly^ | 8,322 | 8,387 | 66 |  |  | 3 | H |
| tRNA^His^ | 8,393 | 8,456 | 64 |  |  | 5 | H |
| tRNA^Cys^ | 8,466 | 8,529 | 64 |  |  | 9 | H |
| tRNA^Gln^ | 8,580 | 8,640 | 61 |  |  | 50 | L |
| tRNA^Leu^ | 8,647 | 8,710 | 64 |  |  | 6 | L |
| *atp8* | 8,729 | 8,884 | 156 | ATG | TAA | 18 | L |
| tRNA^Asn^ | 8,886 | 8,952 | 67 |  |  | 1 | L |
| *atp6* | 8,962 | 9,627 | 666 | ATG | TAA | 9 | L |
| tRNA^Arg^ | 9,628 | 9,692 | 65 |  |  | 0 | L |
| tRNA^Glu^ | 9,698 | 9,762 | 65 |  |  | 5 | L |
| 12S rRNA | 9,763 | 10,505 | 743 |  |  | 0 | L |
| tRNA^Met^ | 10,506 | 10,572 | 67 |  |  | 0 | L |
| *nd3* | 10,571 | 10,924 | 354 | ATG | TAA | -2 | L |
| tRNA^Ser^ | 10,953 | 11,018 | 66 |  |  | 28 | L |
| tRNA^Ser^ | 11,019 | 11,079 | 61 |  |  | 0 | H |
| *nd4* | 11,080 | 12,435 | 1,356 | GTG | TAA | 0 | H |
| tRNA^Thr^ | 12,440 | 12,503 | 64 |  |  | 4 | L |
| *cox3* | 12,504 | 13,278 | 775 | ATG | T-- | 0 | L |
| tRNA^Ile^ | 13,333 | 13,398 | 66 |  |  | 54 | H |
| *nd2* | 13,400 | 14,333 | 934 | ATG | T-- | 1 | H |
| tRNA^Lys^ | 14,334 | 14,397 | 64 |  |  | 0 | H |

**Table S8.** Gene structure of the *Verconia nivalis* mitogenome. H or L indicates that the gene is encoded by the heavy or light strand, respectively.

| **Gene** | **Position** | | **Size** | **Codon** | | **Intergenic**  **nucleotide** | **Strand** |
| --- | --- | --- | --- | --- | --- | --- | --- |
|  | From | To |  | Start | Stop |  |  |
| *cox1* | 1 | 1,533 | 1,533 | ATG | TAA | 226 | H |
| tRNA^Val^ | 1,553 | 1,617 | 65 |  |  | 19 | H |
| 16S rRNA | 1,618 | 2,731 | 1,114 |  |  | 0 | H |
| tRNA^Leu^ | 2,732 | 2,798 | 67 |  |  | 0 | H |
| tRNA^Ala^ | 2,795 | 2,861 | 67 |  |  | -4 | H |
| tRNA^Pro^ | 2,870 | 2,934 | 65 |  |  | 8 | H |
| *nd6* | 2,936 | 3,403 | 468 | TTG | TAA | 1 | H |
| *nd5* | 3,444 | 5,069 | 1,626 | ATG | TAG | 40 | H |
| *nd1* | 5,050 | 5,961 | 912 | GTG | TAA | -20 | H |
| tRNA^Tyr^ | 5,966 | 6,020 | 55 |  |  | 4 | H |
| tRNA^Trp^ | 6,027 | 6,093 | 67 |  |  | 6 | H |
| *nd4l* | 6,094 | 6,381 | 288 | TTG | TAA | 0 | H |
| *cytb* | 6,381 | 7,508 | 1,128 | ATG | TAG | -1 | H |
| tRNA^Asp^ | 7,504 | 7,567 | 64 |  |  | -5 | H |
| tRNA^Phe^ | 7,568 | 7,634 | 67 |  |  | 0 | H |
| *cox2* | 7,638 | 8,312 | 675 | ATG | TAG | 3 | H |
| tRNA^Gly^ | 8,315 | 8,379 | 65 |  |  | 2 | H |
| tRNA^His^ | 8,380 | 8,447 | 68 |  |  | 0 | H |
| tRNA^Cys^ | 8,454 | 8,520 | 67 |  |  | 6 | H |
| tRNA^Gln^ | 8,550 | 8,610 | 61 |  |  | 29 | L |
| tRNA^Leu^ | 8,620 | 8,682 | 63 |  |  | 9 | L |
| *atp8* | 8,684 | 8,836 | 153 | ATG | TAA | 1 | L |
| tRNA^Asn^ | 8,838 | 8,905 | 68 |  |  | 1 | L |
| *atp6* | 8,920 | 9,591 | 672 | ATG | TAA | 14 | L |
| tRNA^Arg^ | 9,592 | 9,657 | 66 |  |  | 0 | L |
| tRNA^Glu^ | 9,658 | 9,720 | 63 |  |  | 0 | L |
| 12S rRNA | 9,721 | 10,468 | 748 |  |  | 0 | L |
| tRNA^Met^ | 10,469 | 10,532 | 64 |  |  | 0 | L |
| *nd3* | 10,531 | 10,884 | 354 | ATG | TAA | -2 | L |
| tRNA^Ser^ | 10,921 | 10,978 | 58 |  |  | 36 | L |
| tRNA^Ser^ | 10,983 | 11,043 | 61 |  |  | 4 | H |
| *nd4* | 11,045 | 12,355 | 1,311 | ATG | TAA | 1 | H |
| tRNA^Thr^ | 12,406 | 12,472 | 67 |  |  | 50 | L |
| *cox3* | 12,473 | 13,247 | 775 | ATG | T-- | 0 | L |
| tRNA^Ile^ | 13,301 | 13,366 | 66 |  |  | 53 | H |
| *nd2* | 13,367 | 14,299 | 933 | GTG | TAA | 0 | H |
| tRNA^Lys^ | 14,309 | 14,369 | 61 |  |  | 9 | H |

**Table S9.** Base composition and skewness of dorid nudibranch mitogenomes.

| **GenBank ID** | **Family** | **Species** | **Size (bp)** | **A%** | **C%** | **G%** | **T%** | **AT%** | **GC%** | **A-T skew** | **G-C skew** |
| --- | --- | --- | --- | --- | --- | --- | --- | --- | --- | --- | --- |
| MT919638 | Cadlinidae | *Aldisa cooperi* | 14,517 | 31.5 | 14 | 16.3 | 38.2 | 69.7 | 30.3 | -0.096 | 0.076 |
| MT919639 | Cadlinidae | *Cadlina japonica* | 14,982 | 29.2 | 14.3 | 18.6 | 37.9 | 67.1 | 32.9 | -0.130 | 0.131 |
| MT919640 | Cadlinidae | *Cadlina koreana* | 14,707 | 31.4 | 13 | 16.5 | 39.1 | 70.5 | 29.5 | -0.109 | 0.119 |
| MT919641 | Cadlinidae | *Cadlina umiushi* | 14,731 | 31.6 | 12 | 16.3 | 40.1 | 71.7 | 28.3 | -0.119 | 0.152 |
| OL800584 | Discodorididae | *Carminodoris armata* | 14,425 | 29.2 | 17.6 | 18.1 | 35.1 | 64.3 | 35.7 | -0.092 | 0.014 |
| OL800585 | Dorididae | *Doris odhneri* | 14,445 | 30.3 | 16.6 | 16.9 | 36.2 | 66.5 | 33.5 | -0.089 | 0.008 |
| MW387958 | Polyceridae | *Triopha modesta* | 14,397 | 29.7 | 15.8 | 17.6 | 36.9 | 66.6 | 33.4 | -0.108 | 0.054 |
| OL800586 | Chromodorididae | *Verconia nivalis* | 14,595 | 29 | 13.1 | 17.3 | 40.6 | 69.6 | 30.4 | -0.167 | 0.138 |

**Table S10.** Codon usage and relative synonymous codon usage (RSCU) of dorid nudibranch mitogenomes.

| **Codon** | ***Aldisa cooperi*** | | ***Cadlia japonica*** | | ***Cadlina koreana*** | | ***Cadlina umiushi*** | | ***Carminodoris armata*** | | ***Doris odhneri*** | | | ***Triopha modesta*** | | | ***Verconia nivalis*** | | |
| --- | --- | --- | --- | --- | --- | --- | --- | --- | --- | --- | --- | --- | --- | --- | --- | --- | --- | --- | --- |
|  | Count | RSCU | Count | RSCU | Count | RSCU | Count | RSCU | Count | RSCU | Count | RSCU | Count | | RSCU | Count | | RSCU |  |
| UUU(F) | 286 | 1.68 | 278 | 1.64 | 326 | 1.88 | 331 | 1.87 | 243 | 1.44 | 268 | 1.62 | 256 | | 1.53 | 320 | | 1.78 |  |
| UUC(F) | 55 | 0.32 | 60 | 0.36 | 21 | 0.12 | 23 | 0.13 | 95 | 0.56 | 63 | 0.38 | 79 | | 0.47 | 40 | | 0.22 |  |
| UUA(L) | 305 | 3.3 | 263 | 2.76 | 333 | 3.61 | 320 | 3.47 | 229 | 2.39 | 233 | 2.53 | 259 | | 2.8 | 296 | | 3.22 |  |
| UUG(L) | 70 | 0.76 | 93 | 0.98 | 66 | 0.72 | 63 | 0.68 | 68 | 0.71 | 61 | 0.66 | 58 | | 0.63 | 68 | | 0.74 |  |
| CUU(L) | 88 | 0.95 | 122 | 1.28 | 108 | 1.17 | 101 | 1.1 | 118 | 1.23 | 114 | 1.24 | 109 | | 1.18 | 107 | | 1.17 |  |
| CUC(L) | 13 | 0.14 | 15 | 0.16 | 11 | 0.12 | 16 | 0.17 | 29 | 0.3 | 33 | 0.36 | 22 | | 0.24 | 11 | | 0.12 |  |
| CUA(L) | 68 | 0.74 | 59 | 0.62 | 32 | 0.35 | 47 | 0.51 | 95 | 0.99 | 91 | 0.99 | 87 | | 0.94 | 59 | | 0.64 |  |
| CUG(L) | 10 | 0.11 | 20 | 0.21 | 3 | 0.03 | 6 | 0.07 | 35 | 0.37 | 20 | 0.22 | 20 | | 0.22 | 10 | | 0.11 |  |
| AUU(I) | 239 | 1.82 | 224 | 1.78 | 239 | 1.87 | 235 | 1.89 | 175 | 1.5 | 211 | 1.72 | 205 | | 1.67 | 240 | | 1.83 |  |
| AUC(I) | 24 | 0.18 | 28 | 0.22 | 17 | 0.13 | 14 | 0.11 | 59 | 0.5 | 35 | 0.28 | 41 | | 0.33 | 23 | | 0.17 |  |
| AUA(M) | 168 | 1.58 | 130 | 1.33 | 191 | 1.67 | 163 | 1.55 | 124 | 1.26 | 158 | 1.72 | 155 | | 1.58 | 138 | | 1.53 |  |
| AUG(M) | 44 | 0.42 | 66 | 0.67 | 38 | 0.33 | 47 | 0.45 | 73 | 0.74 | 26 | 0.28 | 41 | | 0.42 | 42 | | 0.47 |  |
| GUU(V) | 126 | 1.85 | 106 | 1.55 | 123 | 1.86 | 111 | 1.61 | 94 | 1.49 | 131 | 1.87 | 120 | | 1.74 | 125 | | 1.89 |  |
| GUC(V) | 15 | 0.22 | 22 | 0.32 | 7 | 0.11 | 15 | 0.22 | 30 | 0.48 | 28 | 0.4 | 35 | | 0.51 | 18 | | 0.27 |  |
| GUA(V) | 99 | 1.45 | 101 | 1.48 | 107 | 1.62 | 112 | 1.63 | 89 | 1.41 | 95 | 1.36 | 84 | | 1.22 | 97 | | 1.46 |  |
| GUG(V) | 33 | 0.48 | 44 | 0.64 | 28 | 0.42 | 37 | 0.54 | 39 | 0.62 | 26 | 0.37 | 37 | | 0.54 | 25 | | 0.38 |  |
| UCU(S) | 127 | 2.61 | 119 | 2.5 | 144 | 3.04 | 136 | 2.86 | 95 | 2.01 | 97 | 2.1 | 110 | | 2.37 | 132 | | 2.79 |  |
| UCC(S) | 23 | 0.47 | 25 | 0.52 | 9 | 0.19 | 16 | 0.34 | 34 | 0.72 | 34 | 0.74 | 30 | | 0.65 | 6 | | 0.13 |  |
| UCA(S) | 59 | 1.21 | 48 | 1.01 | 58 | 1.22 | 54 | 1.13 | 69 | 1.46 | 61 | 1.32 | 58 | | 1.25 | 66 | | 1.39 |  |
| UCG(S) | 11 | 0.23 | 20 | 0.42 | 6 | 0.13 | 11 | 0.23 | 12 | 0.25 | 12 | 0.26 | 8 | | 0.17 | 5 | | 0.11 |  |
| CCU(P) | 88 | 2.51 | 83 | 2.29 | 87 | 2.5 | 85 | 2.33 | 61 | 1.67 | 73 | 2.04 | 86 | | 2.37 | 96 | | 2.78 |  |
| CCC(P) | 12 | 0.34 | 11 | 0.3 | 6 | 0.17 | 8 | 0.22 | 35 | 0.96 | 24 | 0.67 | 18 | | 0.5 | 6 | | 0.17 |  |
| CCA(P) | 34 | 0.97 | 39 | 1.08 | 45 | 1.29 | 44 | 1.21 | 30 | 0.82 | 40 | 1.12 | 25 | | 0.69 | 30 | | 0.87 |  |
| CCG(P) | 6 | 0.17 | 12 | 0.33 | 1 | 0.03 | 9 | 0.25 | 20 | 0.55 | 6 | 0.17 | 16 | | 0.44 | 6 | | 0.17 |  |
| ACU(T) | 83 | 2.17 | 100 | 2.45 | 102 | 2.62 | 96 | 2.4 | 81 | 1.75 | 87 | 1.84 | 92 | | 2.03 | 100 | | 2.55 |  |
| ACC(T) | 18 | 0.47 | 17 | 0.42 | 2 | 0.05 | 16 | 0.4 | 31 | 0.67 | 28 | 0.59 | 21 | | 0.46 | 16 | | 0.41 |  |
| ACA(T) | 45 | 1.18 | 35 | 0.86 | 48 | 1.23 | 40 | 1 | 60 | 1.3 | 64 | 1.35 | 61 | | 1.35 | 31 | | 0.79 |  |
| ACG(T) | 7 | 0.18 | 11 | 0.27 | 4 | 0.1 | 8 | 0.2 | 13 | 0.28 | 10 | 0.21 | 7 | | 0.15 | 10 | | 0.25 |  |
| GCU(A) | 106 | 2.04 | 115 | 2.14 | 130 | 2.61 | 126 | 2.52 | 115 | 1.84 | 129 | 2 | 111 | | 1.96 | 135 | | 2.61 |  |
| GCC(A) | 27 | 0.52 | 35 | 0.65 | 13 | 0.26 | 14 | 0.28 | 59 | 0.94 | 50 | 0.78 | 35 | | 0.62 | 17 | | 0.33 |  |
| GCA(A) | 61 | 1.17 | 35 | 0.65 | 51 | 1.03 | 57 | 1.14 | 61 | 0.98 | 65 | 1.01 | 59 | | 1.04 | 52 | | 1 |  |
| GCG(A) | 14 | 0.27 | 30 | 0.56 | 5 | 0.1 | 3 | 0.06 | 15 | 0.24 | 14 | 0.22 | 22 | | 0.39 | 3 | | 0.06 |  |
| UAU(Y) | 86 | 1.35 | 96 | 1.55 | 113 | 1.78 | 107 | 1.71 | 79 | 1.22 | 102 | 1.5 | 89 | | 1.35 | 103 | | 1.53 |  |
| UAC(Y) | 41 | 0.65 | 28 | 0.45 | 14 | 0.22 | 18 | 0.29 | 51 | 0.78 | 34 | 0.5 | 43 | | 0.65 | 32 | | 0.47 |  |
| UAA(*) | 0 | 0 | 0 | 0 | 0 | 0 | 0 | 0 | 0 | 0 | 0 | 0 | 0 | | 0 | 0 | | 0 |  |
| UAG(*) | 0 | 0 | 0 | 0 | 0 | 0 | 0 | 0 | 0 | 0 | 0 | 0 | 0 | | 0 | 0 | | 0 |  |
| CAU(H) | 63 | 1.68 | 63 | 1.7 | 63 | 1.75 | 68 | 1.84 | 53 | 1.43 | 48 | 1.3 | 64 | | 1.73 | 65 | | 1.73 |  |
| CAC(H) | 12 | 0.32 | 11 | 0.3 | 9 | 0.25 | 6 | 0.16 | 21 | 0.57 | 26 | 0.7 | 10 | | 0.27 | 10 | | 0.27 |  |
| CAA(Q) | 38 | 1.49 | 33 | 1.22 | 44 | 1.54 | 38 | 1.33 | 34 | 1.24 | 40 | 1.43 | 40 | | 1.51 | 37 | | 1.48 |  |
| CAG(Q) | 13 | 0.51 | 21 | 0.78 | 13 | 0.46 | 19 | 0.67 | 21 | 0.76 | 16 | 0.57 | 13 | | 0.49 | 13 | | 0.52 |  |
| AAU(N) | 93 | 1.68 | 87 | 1.63 | 114 | 1.88 | 102 | 1.81 | 58 | 1.18 | 75 | 1.44 | 75 | | 1.4 | 89 | | 1.68 |  |
| AAC(N) | 18 | 0.32 | 20 | 0.37 | 7 | 0.12 | 11 | 0.19 | 40 | 0.82 | 29 | 0.56 | 32 | | 0.6 | 17 | | 0.32 |  |
| AAA(K) | 76 | 1.58 | 68 | 1.42 | 82 | 1.64 | 76 | 1.65 | 68 | 1.45 | 79 | 1.66 | 72 | | 1.53 | 66 | | 1.39 |  |
| AAG(K) | 20 | 0.42 | 28 | 0.58 | 18 | 0.36 | 16 | 0.35 | 26 | 0.55 | 16 | 0.34 | 22 | | 0.47 | 29 | | 0.61 |  |
| GAU(D) | 58 | 1.78 | 52 | 1.55 | 56 | 1.75 | 61 | 1.88 | 42 | 1.25 | 51 | 1.48 | 47 | | 1.32 | 58 | | 1.66 |  |
| GAC(D) | 7 | 0.22 | 15 | 0.45 | 8 | 0.25 | 4 | 0.12 | 25 | 0.75 | 18 | 0.52 | 24 | | 0.68 | 12 | | 0.34 |  |
| GAA(E) | 70 | 1.47 | 68 | 1.48 | 74 | 1.63 | 72 | 1.62 | 52 | 1.18 | 67 | 1.49 | 70 | | 1.59 | 63 | | 1.3 |  |
| GAG(E) | 25 | 0.53 | 24 | 0.52 | 17 | 0.37 | 17 | 0.38 | 36 | 0.82 | 23 | 0.51 | 18 | | 0.41 | 34 | | 0.7 |  |
| UGU(C) | 51 | 1.82 | 53 | 1.83 | 49 | 1.81 | 50 | 1.82 | 39 | 1.5 | 40 | 1.48 | 50 | | 1.72 | 57 | | 1.87 |  |
| UGC(C) | 5 | 0.18 | 5 | 0.17 | 5 | 0.19 | 5 | 0.18 | 13 | 0.5 | 14 | 0.52 | 8 | | 0.28 | 4 | | 0.13 |  |
| UGA(W) | 70 | 1.52 | 53 | 1.13 | 67 | 1.41 | 73 | 1.57 | 57 | 1.24 | 63 | 1.34 | 67 | | 1.44 | 71 | | 1.48 |  |
| UGG(W) | 22 | 0.48 | 41 | 0.87 | 28 | 0.59 | 20 | 0.43 | 35 | 0.76 | 31 | 0.66 | 26 | | 0.56 | 25 | | 0.52 |  |
| CGU(R) | 23 | 1.7 | 18 | 1.36 | 14 | 1.08 | 15 | 1.13 | 15 | 1.11 | 19 | 1.33 | 13 | | 0.95 | 30 | | 2.22 |  |
| CGC(R) | 2 | 0.15 | 3 | 0.23 | 0 | 0 | 2 | 0.15 | 2 | 0.15 | 2 | 0.14 | 4 | | 0.29 | 0 | | 0 |  |
| CGA(R) | 22 | 1.63 | 19 | 1.43 | 19 | 1.46 | 25 | 1.89 | 20 | 1.48 | 26 | 1.82 | 24 | | 1.75 | 18 | | 1.33 |  |
| CGG(R) | 7 | 0.52 | 13 | 0.98 | 19 | 1.46 | 11 | 0.83 | 17 | 1.26 | 10 | 0.7 | 14 | | 1.02 | 6 | | 0.44 |  |
| AGU(S) | 59 | 1.21 | 51 | 1.07 | 53 | 1.12 | 40 | 0.84 | 51 | 1.08 | 62 | 1.34 | 46 | | 0.99 | 68 | | 1.44 |  |
| AGC(S) | 15 | 0.31 | 10 | 0.21 | 8 | 0.17 | 7 | 0.15 | 27 | 0.57 | 11 | 0.24 | 12 | | 0.26 | 13 | | 0.27 |  |
| AGA(S) | 84 | 1.72 | 71 | 1.49 | 83 | 1.75 | 96 | 2.02 | 65 | 1.38 | 72 | 1.56 | 84 | | 1.81 | 77 | | 1.63 |  |
| AGG(S) | 12 | 0.25 | 37 | 0.78 | 18 | 0.38 | 21 | 0.44 | 25 | 0.53 | 20 | 0.43 | 24 | | 0.52 | 12 | | 0.25 |  |
| GGU(G) | 65 | 1.05 | 75 | 1.16 | 71 | 1.15 | 61 | 0.95 | 57 | 0.93 | 54 | 0.91 | 77 | | 1.17 | 110 | | 1.71 |  |
| GGC(G) | 15 | 0.24 | 10 | 0.16 | 8 | 0.13 | 15 | 0.23 | 19 | 0.31 | 15 | 0.25 | 15 | | 0.23 | 10 | | 0.16 |  |
| GGA(G) | 117 | 1.89 | 107 | 1.66 | 129 | 2.1 | 146 | 2.28 | 96 | 1.57 | 114 | 1.92 | 105 | | 1.6 | 101 | | 1.57 |  |
| GGG(G) | 50 | 0.81 | 66 | 1.02 | 38 | 0.62 | 34 | 0.53 | 72 | 1.18 | 54 | 0.91 | 66 | | 1 | 37 | | 0.57 |  |

**Table S11.** Nudibranch species collected and used for mitogenome sequencing in the present study.

| **Family** | **Species** | **Locality** |
| --- | --- | --- |
|  |  |  |
| Cadlinidae | *Aldisa cooperi* | Munamjin-ri, Gangwon-do, South Korea |
| Cadlinidae | *Cadlina japonica* | Munamjin-ri, Gangwon-do, South Korea; |
| Cadlinidae | *Cadlina koreana* | Munamjin-ri Gangwon-do, South Korea |
| Cadlinidae | *Cadlina umiushi* | Munamjin-ri, Gangwon-do, South Korea |
| Discodorididae | *Carminodoris armata* | Tongyeong, Gyeongsangnam-do, South Korea |
| Dorididae | *Doris odhneri* | Munamjin-ri Gangwon-do, South Korea |
| Polyceridae | *Triopha modesta* | Hyeonnam-myeon, Gangwon-do, South Korea |
| Chromodorididae | *Verconia nivalis* | Chawido, Jeju-do, South Korea |
